# Spatial multiomics identifies rheumatoid arthritis synovial niches and *de-novo* laminar histological patterns of fibroblasts subtypes

**DOI:** 10.3389/fimmu.2026.1883263

**Published:** 2026-10-16

**Authors:** Sílvia Sisó, Archana Iyer, Vineeth Raghuram, Neha Chaudhary, Heather Knight, Fei Wang, Ioan Filip, Lori Duggan, Xue Wang, Katherine Naughton, Terry Melim, Anna Yarilina, Yingli Yang, Jocelyn Rivas, Abby McCarthy, Yu Tian, Dan Chang, Timothy R.D.J. Radstake, Melanie C. Ruzek, Annette Schwartz-Sterman

**Affiliations:** 1 Pathology Department, Immunology Discovery Research, AbbVie Bioresearch Center, Worcester, MA, United States; 2 Immunology Discovery Research, AbbVie, Cambridge Research Center, Cambridge, MA, United States; 3 Immunology Discovery Research, Research & Development, AbbVie Inc., North Chicago, IL, United States

**Keywords:** healthy, histology, rheumatoid arthritis, spatial multiomics, spatial proteomics, spatial transcriptomics, synovial fibroblasts, synovium

## Abstract

**Introduction:**

Spatial omic technologies provide insight into molecular architecture and spatial cellular organization within tissue. In rheumatoid arthritis (RA), single-cell RNA sequencing and low-resolution spatial transcriptomics have advanced the understanding of synovial biology. However, single-cell spatial resolution using multiple spatial platforms to comprehensively map the immune and stromal cell organization as well as topographical cellular interactions in the RA synovium remain limited.

**Methods:**

We integrated single-cell and bulk spatial transcriptomics (CosMx and Xenium, and Visium, respectively) as well as spatial LC/MS proteomics and a multiplex immunohistochemical protein panel in the histological evaluation of a non-clinical cohort of six RA, three osteoarthritis (OA) and three healthy (NH) arthroscopic synovial biopsies. The histopathological observations were integrated with Xenium-based spatial computational analyses in the RA and NH synovium to define fibroblast organization and spatial relationships in RA compared to the NH synovium.

**Results:**

Cross-platform comparisons demonstrated a strong concordance in cellular composition and microanatomical localization and highlighted the strengths of each technology, with single-cell spatial analysis providing higher cellular resolution, but reduced molecular depth. Comparison of single-cell spatial transcriptomics and LC/MS proteomics also demonstrated concordance, but revealed region-specific expression of fibroblast-associated genes and proteins. Spatial mapping in our large biopsies identified a previously unrecognized laminar organization of up to nine fibroblast subtypes spanning the lining and sublining regions of RA synovium, distinct from NH. Spatial computational pathology analyses showed that specific fibroblast subtypes preferentially localized within lymphoid (CD74+ and CXCL12+), vascular (NOTCH3+ and mural) and stromal (POSTN+, DKK3+ and CD34+) niches. Pathway enrichment analyses further implicated specific biological functions to the fibroblast subtypes that were linked to their microenvironment.

**Conclusion:**

This multimodal spatial molecular pathology study indicates that in RA, fibroblast subtypes expand, spatially reorganize, and establish specialized tissue niches that likely contribute to disease pathogenesis. These findings provide a high-resolution spatial framework and support future spatial multiomics studies aimed at identifying fibroblast ecotypes in the context of treatment naïve, treatment-responsive and treatment-refractory RA patients to identify tissue-level spatial signatures associated with inadequate response to treatments.

## 1 Introduction

Rheumatoid arthritis (RA) is a debilitating autoimmune disease characterized by inflammation and neovascularization in the synovium of joints, resulting in severe pain and eventual destruction of joint cartilage and bone. Histologically, the healthy synovium consists of an intimal lining layer that is 1–2 cells thick composed of type A and type B fibrocytes, corresponding to specialized macrophages and fibroblasts, respectively; and a synovial sublining layer (or subintima) containing fibrous, areolar and/or adipose tissue (1, 2). In some joints, the subintima of the synovium may consist of periosteum, perimysium, or even hyaline cartilage or fibrocartilage (2). The areolar type is the most specialized type, often crimped into folds of villi composed of a continuous layer of cells, a superficial subintimal vascular plexus with capillaries, small arterioles and venules, associated with mast cells, nerve endings and a fine matrix with type I collagen fibers (2, 3).

In RA, there is an enlargement of the synovial lining cell layer, increased density of the synovial stroma, and inflammatory infiltrates. Histopathological evaluation of inflammation from RA synovial specimens can include the Krenn synovitis score based on severity (4), as well as ‘pathotype’ based on predominant cell type (the lympho-myeloid dominated by B cells and macrophages; the diffuse-myeloid with predominant myeloid cells but B cell poor; and fibroid pauci-immune with scanty immune cells and prevalent stromal cells) (5).

Building on the histopathological signature of RA, several molecular profiling studies of synovial tissue have furthered our understanding of cellular composition in the RA synovium and potential drivers of disease using bulk RNAseq, single-cell RNAseq, mass cytometry and proteomic analyses (6–12). In particular, the Accelerating Medicines Partnership (AMP) consortium has reported cell type abundance phenotypes (CTAP) amongst patients, with enrichment of certain cell type subpopulations, including lymphocytes, myeloid, endothelial, and stromal cells (10). Furthermore, integration of scRNA-seq datasets has defined various subtypes of macrophages and fibroblasts based on distinct gene signatures (10, 13), which in the context of RA pathotypes, may aid the stratification of patients based on clinical severity and response to treatment (5, 14, 15).

The rapid emergence of new spatial biology technologies which allow for the detection of larger numbers of proteins or/and transcripts at the subcellular, cellular or multicellular level in formalin-fixed paraffin-embedded (FFPE) tissues or cryosections (9, 16–22) is starting to be explored in RA (9, 23–26). Single-cell spatial transcriptomics (scST) approaches are already advancing our understanding of cellular and molecular drivers of RA patients in remission and for those experiencing treatment resistance in early OA (24, 25, 27). Thus, the application of multimodal spatial omic approaches provides not only a better understanding of the intricate cellular interactions in the disease state but also has the potential to accelerate drug and biomarker discovery (28–30).

Success in this area of spatial biology requires a transition towards pathology laboratories adopting these novel spatial histomolecular technologies (31) and building robust computational frameworks that transform multimodal imaging data into spatial transcriptomics datasets that align with pre-existing single-cell/bulk RNA seq data (32, 33). This paper first describes the laboratory workflow as well as various quality and verification computational processes of a multimodal spatial omic study on healthy, RA and OA synovium. The technical study explores three spatial proteomics assays (laser capture microscopy combined with LC/MS proteomics, multiplex immunofluorescence and chromogenic immunohistochemistry), and three spatial transcriptomics modalities encompassing mini-bulk sequencing based whole-transcriptome transcriptomics and single-cell imaging based targeted transcriptomics (31, 34) in a non-clinical cohort of 12 human synovial samples to explore the advantages of each transcriptional profiling modality.

Through the histological assessment of major synovial compartments and the topography of major cell populations, distinct patterns in the fibroblast gene and protein expression were observed, leading to a second, multimodal in-depth analysis of fibroblast subtype localization, organization and functional comparisons between healthy and RA synovium through combining single-cell spatial transcriptomics and proteomics with histology.

This spatial multiomic study provides a foundation for establishing successful spatial biology pipelines in molecular pathology laboratories, and for the first time, unveils the spatial histomolecular architecture of RA synovial fibroblasts *in situ* in comparison to healthy synovium.

## 2 Materials and methods

### 2.1 Human synovial samples, histology-based quality control, and evaluation of pathotypes

Synovial formalin-fixed paraffin-embedded (FFPE) blocks including 6 RA (RA1-RA6), 3 osteoarthritis (OA1-OA3), and 3 healthy (NH1-NH3) were procured from commercial vendors as detailed in Wang et al. (35) and subjected to downstream spatial omics investigations. The only clinical metadata available for these samples were demographics. Inclusive criteria for spatial omic studies were based on three major quality control steps: i) good tissue size and cellularity, ii) good histological morphology with representation of the synovial lining (SL) and the synovial sublining (SSL) on H&E-stained tissue sections, and iii) good quality protein and mRNA based on chromogenic immunohistochemistry (IHC) for various proteins and *in situ* hybridization (ISH) for peptidylprolyl isomerase B *(PPIB)*, respectively. Briefly, FFPE blocks were sectioned at 5 µm in Diethyl pyrocarbonate (DEPC)-treated water and stained with hematoxylin-eosin (H&E). Additional sections were processed for IHC to assign a pathotype according to the Pitzalis’ RA classification criteria (36). Immunohistochemistry was performed to detect T cells (CD3, clone SP7; ThermoScientific, Cat #RM-9107-S), B-cells (CD19, Clone BT51E; Leica, Cat #PA0843), macrophages (CD68, Clone SP251; Abcam, Cat #ab192847), plasma cells (CD138, Clone SP152; Abcam, Cat #ab130405), and fibroblasts (Podoplanin, Clone D2-40; Biolegend, Cat #916606), (CD90/Thy1, Clone EPR3132; Abcam, Cat #ab92574), (PDGF Receptor ß, Clone 28E1; Cell Signaling Technology, Cat #3169) and (TEM1/CD248, Clone B1/35; BD Pharmingen, Cat #564993)], using the Leica Bond RX automated immunostainer (Leica Biosystems, Deer Park, IL) as previously described (35). To assess the mRNA quality of the procured synovial FFPE blocks, *in situ* hybridization was performed on the Leica Bond RX using Advanced Cell Diagnostic’s (ACD) RNAscope 2.5 LSx assay. ACD’s 2.5 LSx assay incorporates a series of proprietary reagents, the positive control probe, *PPIB* (ACD/Bio-Techne, Newark, CA; Cat # 313908) and the negative control probe, dapB (ACD, Cat # 312038). Visualization was done with either RNAscope brown LS reagent kit (ACD, Cat #322100) or the red LS reagent kit (ACD, Cat #322150). All slides were scanned on the Pannoramic 250 whole slide digital scanner (3DHISTECH Ltd, Budapest, Hungary) using either a 20x or 40x lens with extended focus. Quality of mRNA from all FFPE samples was semi-quantitatively evaluated by a trained pathologist in multiple high-power fields for frequency of PPIB positive cells with the following criteria: 0, no + cells; 1, rare cells, focal; 2, 5-10% of cells, multifocal; 3, 20-40% of cells, diffuse (35). All selected subjects had *PPIB* scores of 2 or above.

Success in spatial omics studies requires a transition towards pathology laboratories building robust bench workflows and computational frameworks that transform multimodal imaging data into spatial transcriptomics datasets.

### 2.2 Histological bench workflow for spatial omic investigations

For each arthroscopic synovial sample, a pathologist annotated a single 6mm-in-diameter ROI that best represented histopathological features of the disease, i.e., synovial hyperplasia, lymphoid aggregates, vascular proliferation, and adipose tissue replacement with fibrous stroma. **Supplementary Figures 1A, B** provides a histological overview of all ROI from healthy, OA or RA patients. To be able to build spatial omics glass slides, H&E pre-selected ROIs were scored in their corresponding FFPE block using a 6mm diameter punch device (Integra Miltex; Integra LifeSciences, Mansfield, MA), sectioned in the microtome at 5 µm thickness and transferred to a glass slide (**Figure 1B**). Notably, spatial omic platforms (i.e., Visium, Xenium, LCM proteomics) require specific types of glass slides to be used as well as restrictions of glass area that can be filled with tissue sections. This procedure was repeated for each FFPE block and spatial omic platform, allowing for three different patient samples to be placed on the same glass slide.

**Figure 1:**
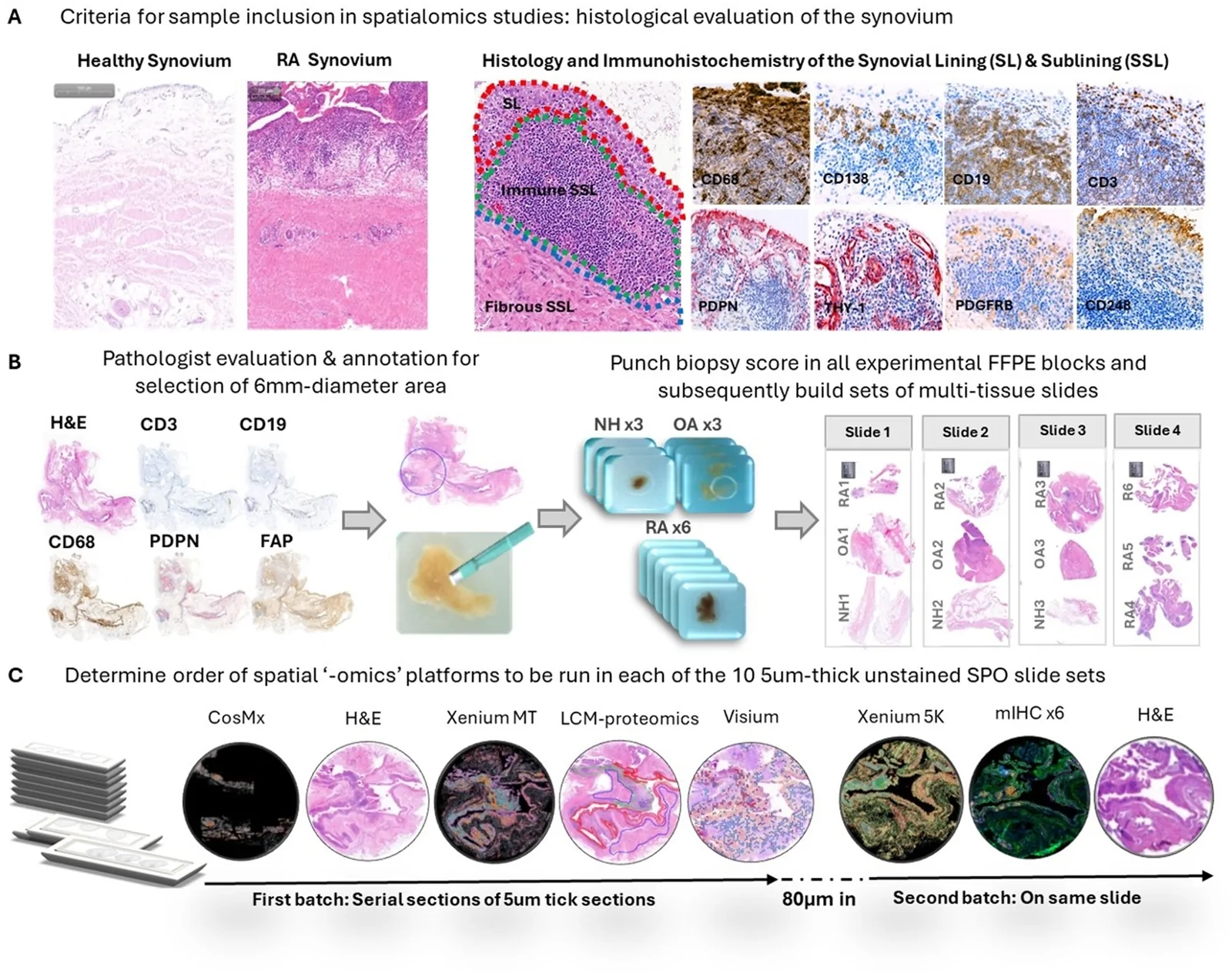
Histological practices for experimental spatial omics. **(A)** Criteria for including samples for downstream spatial multiomics work includes morphological features such as synovial lining and sublining based on H&E evaluation and immune IHC markers (CD68, CD138, CD19 and CD3) as well as fibroblast markers (PDPN, THY1, PDGFRb and CD248). **(B)** Selection of areas with representative RA histopathology are selected as a ROI of 6mm in diameter on the FFPE block. Punch biopsies for all experimental samples are scored for each block, and cut sections are placed onto glass slides. **(C)** Replicates of the unstained sections on glass slides are run for various platforms either as batches of serial samples or on the same sample/slide. The H&E is performed on each batch for co-registration purposes.

For this technical work, 12 synovial samples were distributed in 4 glass slides, and 10 serial replicates from each glass slide were generated (**Figures 1B, C**) and stored in a desiccator at room temperature until use. The first and second slide replicates were dedicated to CosMx (Bruker, Billerica, MA) using their 1K gene panel, the third slide was stained with H&E, the fourth and fifth replicates were used for Xenium using the Human MultiTissue-Custom panel composed of 377 genes and 40 custom genes added on (10X Genomics, Pleasanton, CA), the sixth and seventh for LCM-proteomics and the remaining eight and ninth slides were used for Visium (10X Genomics). The extra-slides sectioned for each platform were secured in case it needed to be repeated. Subsequently, two additional multiomics slides including NH3 and RA3-RA6 were processed sequentially in Xenium and by multiplex immunofluorescence (mIF) IHC using the Xenium Prime 5K Human Pan Tissue panel (10X Genomics) and the PhenoCode Signature Immune Profile Human Protein Panel (Akoya Biosciences, Marlborough, MA, Cat #PCSPP002), respectively. An illustrative summary of the complete spatial omic bench pipeline performed in these 12 samples is shown in **Supplementary Figure 1B**. Details of each spatial omic technology’s assay protocol can be found as **Supplementary Materials and Methods**.

### 2.3 Spatial omic computational framework for transforming multimodal imaging data

#### 2.3.1 Visium data filtering and deconvolution

The image data and the fastq files were processed using the Space ranger pipeline (version 2.1.1) provided by 10x to generate the output files, which could be imported into Seurat (version 4) (37) for analysis. Spots with less than 500 genes were filtered out. The filtered individual Seurat objects were normalized using SCTransform function. Spot deconvolution was run on the samples individually samples using robust cell type decomposition (RCTD) (38) software package runs in full mode which assigns probabilities to each spot in the data for all cell types in the reference. The reference used was the AMP Phase 2 RA dataset with 11 broad cell type annotations.

#### 2.3.2 Cell segmentation

Regarding the cell segmentation approach, CosMx and Xenium both used DAPI based nuclear and cellular boundary, respectively. With regards to the Xenium data, we employed the unimodal Xenium cell segmentation algorithm which employs a heuristic approach to identify the cell nuclei and expand the nuclear boundary 15µm to create the cell boundaries via a Voronoi tessellation methodology. Visual inspection by a trained pathologist of cell boundaries in the H&E post-Xenium stained sections served as quality control of the cell segmentation performance (**Supplementary Data**).

#### 2.3.3 CosMx analysis and cell type annotations

All samples were processed with CosMx 1K plex panel with 24 custom probes (**Supplementary Material**) for Spatial imaging using Nanostring’s CosMx Spatial Molecular Imager (SMI) capturing ~40 FOVs per sample. A reference matrix of synovial cell type expression profiles was constructed using the AMP Phase 2 RA single-cell atlas (10) using SpatialDeconR Library. Supervised cell typing was then performed with Insitutype into 11 major cell types and then subclustered to fine cell types. Cluster probability cutoff of 0.9 was used for major cell types and 0.75 for closely related cell types, resulting in an annotation of 31 cell types (data not shown). Adipocytes were clustered using a single R package. The Seurat package (v4.0) was used to perform QC analysis including preprocessing, UMAP, and to identify marker genes for each subcluster (**Supplementary Figure 2C**).

#### 2.3.4 Xenium data analysis and cell type annotations

Xenium output data were processed as per the standard Seurat processing pipeline (37). Briefly, pre-filtering was performed to remove cells with less than 1 transcript and followed by data counts that were normalized using the SCTransform function. Last, dimensionality reduction was performed using PCA, UMAP and Neighbors (dimensions n= 40), and cluster resolution of 0.3. Following data pre-processing, cell type annotations were performed on a single-cell level using the SingleR package (39), with the AMP Phase 2 RA dataset for 11 broad and 42 extended annotations. Following cell type annotation, QC diagnostics were performed by evaluating the confidence score heatmap comparing query and reference cell type profiles, the top 10 marker genes for major cell types and subtypes. Alignment of the H&E images and mIF-IHC to the Xenium DAPI image, followed by integration of the cell type annotations, was performed in the Xenium Explorer package. Additional QC was performed by histological evaluation of the Xenium cell types overlaid with the corresponding mIF-IHC (**Figure 2A**) and H&E images.

**Figure 2:**
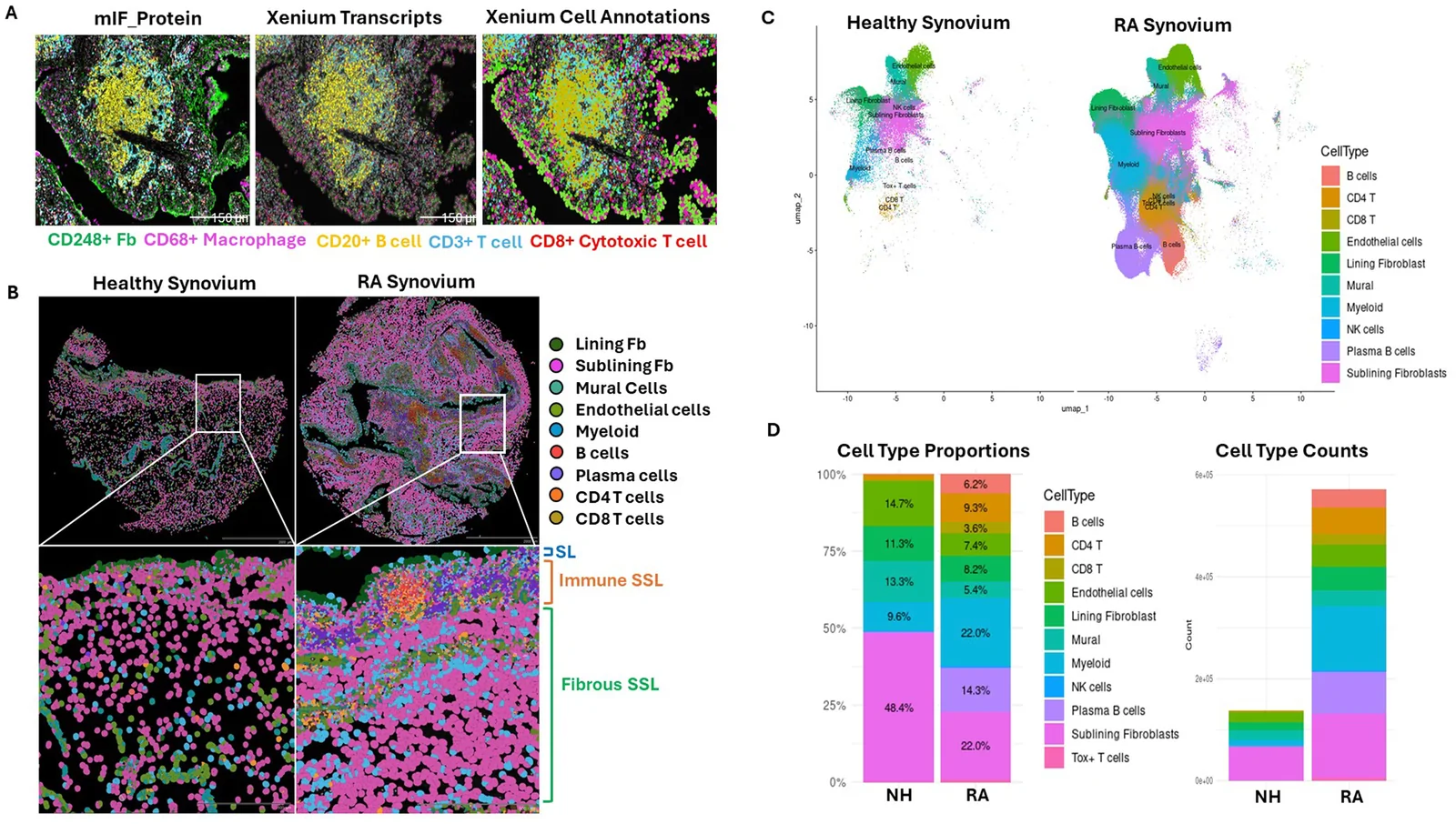
Verification of single-cell RNA sequencing cell type annotations with single-cell spatial transcriptomics and assessment of cellular composition in healthy and diseased synovial tissue. **(A)** Multiplex immunofluorescence immunohistochemistry, Xenium transcripts and cell annotations are overlayed on the same section for CD248 (fibroblasts), CD68 (macrophages), CD3 (T cells), CD8 (cytotoxic T cells) and CD20 (B cells). **(B)** Cell type annotations from the Xenium MultiTissue-Custom panel highlighting the topographical distribution of major cell types in the healthy (NH3) and RA (RA3) synovium. Specific synovial compartments (synovial lining [SL], and immune and fibrous synovial sublining (SSL) are annotated in the RA synovium. **(C)** UMAP of major cell types in NH, and RA synovium from Xenium using the MultiTissue- Custom gene panel. **(D)** Cell type proportions (left) and total cell counts (right) of broad cell types.

#### 2.3.5 Spatial omic data visualization and preliminary analysis in bioturing

Raw Xenium and CosMx output data, as well as metadata corresponding to cell type annotations, acquisition FOVs, Niche ROIs, LCM annotations, disease status, were loaded into the Bioturing ^©^ cloud platform for further visualization and plotting within the single-cell lens module created with [BBrowserX/BBrowserX Pro/Talk2Data/BioTuring Lens/SpatialX] software (BioTuring Inc., San Diego, CA, USA). The bubble plot analysis functionality was used to generate relative gene expression profiles for selecting fibroblast marker gene signatures across platforms, disease conditions, cell types, and tissue sub-compartments.

#### 2.3.6 Comparison of major cell type proportions and differential gene expression between xenium, CosMx and visium

To compare the cell type counts across technologies, we manually annotated all selected CosMx FOVs (**Supplementary Figure 1**) and transferred them as ROIs to all Xenium and Visium samples. To compare the major cell type proportions inferred from Xenium and CosMx technologies, we first created a new single-cell reference by merging the DMARD naive cohort from AMP-RA (10) with dataset GSE216651 (40), which contained 4 healthy donors and 5 OA patients. Thus, we created a more balanced reference across healthy controls, OA and RA, with the addition of “Adipose cells” as a new cell type that was not captured in the AMP-RA dataset. We then created pseudobulk expression profiles for the major cell types in our merged reference, using the Aggregate Expression function in Seurat v5.0: Adipose cells, B-cells, CD4 T cells, CD8 T cells, Endothelial cells, Lining Fibroblasts, Sublining Fibroblasts, Mural cells, Myeloid cells, NK cells and Plasma cells (**Supplementary Table 1**). We then used SingleR (39) and Insitutype (41) to transfer the major cell type labels (excluding adipose cells and NK cells) to our Xenium and CosMx samples respectively, in a supervised fashion. We obtained similarly consistent cell type proportions when using SingleR for CosMx data instead of Insitutype, but we opted for Insitutype-based annotations in CosMx, per NanoString materials (41). To compare these proportions with the Visium RCTD results, we calculated the average of the proportions for the 11 broad cell types across each section and compared them to CosMx and Xenium cell type proportions mentioned above. Inferred cell type proportions were then compared between CosMx and their manually matched FOVs in Xenium and Visium (see **Figure 3A**; **Supplementary Figures 2A, B**).

**Figure 3:**
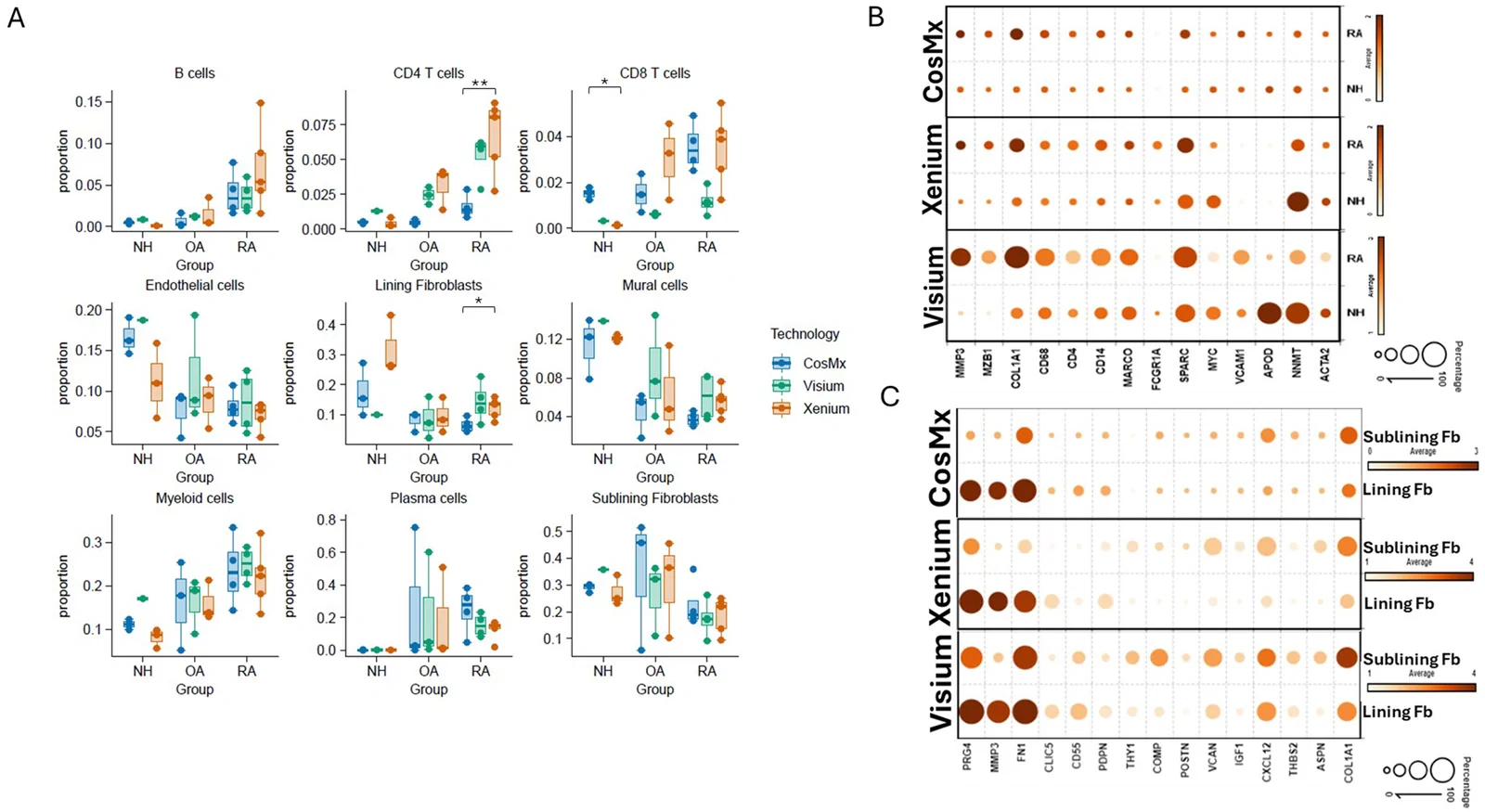
Comparison of gene expression patterns detected by spatial transcriptomics platforms in non-overlapping ROIs. **(A)** Cell type proportions detected by CosMx, Visium and Xenium (MultiTissue panel) platforms highlighting concordant data. Lymphocyte and myeloid cell proportions dramatically increased in RA vs healthy, while endothelial cells and fibroblasts show the reverse trend, proportionally decreased in RA vs healthy. When comparing the two scST platforms, most comparisons were not significant by Wilcoxon test, after adjusting for multiple hypotheses. However, some notable exceptions included consistently higher proportions of *CD4+* T cells and lining fibroblasts in RA from Xenium and higher proportions of *CD8+* T cells in the healthy synovium from CosMx (asterisks: **p < 0.01, *p < 0.05). **(B, C)** Top differentially expressed fibroblast genes using AMP2 datasets as a reference verified across three spatial transcriptomic platforms, Visium as a mini-bulk approach in spots with sufficient UMIs, CosMx as a single-cell resolution but in restricted ROIs, and Xenium as a single-cell resolution approach on the whole section (see **Supplementary Figure 1**). **(B)** Differential gene expression between healthy (NH) and RA synovial samples. **(C)** Differential gene expression in all (NH and RA) samples when comparing the synovial lining and sublining.

Due to the difference in technologies and their sensitivity, it is not possible to compare the expressions directly. Therefore, to assess the concordance of gene expression across spatial transcriptomics platforms, we analyzed differences in gene expression between diseased and healthy samples within each technology. We performed differential gene expression analysis comparing RA and NH cells/spots across the entire sample. For genes identified as differentially expressed by any two technologies, we calculated the Pearson correlation coefficient based on log2 fold changes between disease and healthysamples. In all three pairwise comparisons, we observed a robust correlation of 0.75 or higher.

#### 2.3.7 LCM -proteomics correlation with xenium data

To compare mRNA to protein expression on 29 overlapping fibroblast marker features from the same synovial compartments in NH and RA synovia, Xenium spatial transcriptomics and laser capture microdissection (LCM) spatial proteomics were analyzed on the same ROIs from serial FFPE sections. The methods and data analysis of LCM spatial proteomics were described in our previous publication (35). For Xenium spatial transcriptomics, we quantified the average expression level and expressing cell percentage of each marker gene in each ROI using the Bioturing platform, then we multiplied the average expression level and cell percentage to attain ‘Final Xenium Score’ for each marker gene as the RNA expression indicator. For LCM spatial proteomics, protein expression abundances in each ROI were used as the protein expression indicator. We calculated pairwise log2 fold change among SL and SSL regions in NH synovium membrane and SL, as well as immune SSL and fibrous SSL subdivisions in the RA synovium for both RNA and protein expression indicators of the overlapping 29 fibroblast markers. The results were displayed in 7 pairwise comparison scatter plots. Pearson coefficients are calculated to test the positive correlations by cor.test() function in R and the significance of such correlations was calculated using 20, 000 permutations and a two-sided test.

### 2.4 Leveraging single-cell spatial transcriptomics to bring insights into RA fibroblast topography, cell-to-cell interactions, spatial niches and function

#### 2.4.1 Neighborhood analysis

Pearson correlation of fibroblast cellular neighborhoods was performed on a per-sample basis using the hoodscanR package in R (42) and subsequently averaged across the RA and NH groups. In brief, processed Seurat objects from the Xenium analysis and cell type annotations were converted to spatialExperiment objects, followed by a subsequent nearest neighbor identification using the Approximate Nearest Neighbor (ANN) algorithm using a k = 100 – chosen to effectively capture the morphological microenvironments of the fibroblast cell types. The hoodScanR package identifies neighborhood probabilities using the SoftMax function and are followed by neighborhood colocalization analysis which computes the Pearson correlation of the neighborhoods of cells.

#### 2.4.2 Spatial cellular interactions scores

Spatial interaction scores across RA and NH samples between all cell types of interest were performed using the spatial interaction function from the scimap package (43). Processed xenium data were converted to h5AD (annData) objects, followed by the scimap analysis pipeline in Python for spatial interactions. In brief, the spatial interaction function calculates the likelihood (probability) of cell types being adjacent to each other, compared to a permuted background (1000 permutations). Neighborhood identification was performed using the k-nearest-neighbors (knn), with k = 100 to match the methodology used for the Pearson correlation method noted above. The resulting heatmaps display the significant interaction probabilities (p-value: 0.05) of select cell types averaged across all samples in the healthyand RA categories. Red and blue shading indicates increased and likelihood of interactions, while white shaded boxes indicate cells with 0 or insignificant probability of interactions.

#### 2.4.3 Targeted microdomain/niche analyses

B cell ROIs were identified on the post-Xenium mIF-IHC Phenocode Signature (PSP) image, using a thresholding algorithm devised in the Visiopharm ^®^ software to identify CD20 hotspots and subsequently expanded to create a series of 15 µm non-overlapping regions up to 75µm. Following this process, the mIF image was aligned with the Xenium DAPI and H&E images (confirming the lymphoid aggregate cluster) where the integration of the AMP-RA annotated cell types with the B Cell ROIs was performed using a custom-built python package. Following this process, the relative abundance of the fibroblast subtypes was quantified within each of the B cell microdomains across the RA and healthy samples, to create a heatmap of the results. Vascular ROIs were drawn by a pathologist and subsequently expanded to a series of 15 µm non-overlapping regions up to 75µm in Visiopharm. ROIs were integrated with Xenium cell type data identical to the process outlined above, and relative cell abundance was calculated for the healthyand RA sample groups separately.

#### 2.4.4 Local Moran’s I analysis

Prior work in glioblastoma have used spatial autocorrelation metrics (e.g Moran’s I) to quantify the extent of disease and the biology of different neuronal cell types (44) – finding changes in the structured and disorganized subregions between disease and healthy regions. Following processing of theXenium spatial data with Seurat and cell type annotations with the SingleR package, we converted the Seurat objects to a SpatialFeatureExperiment object (45) and performed spatial autocorrelation analysis using the spdep package in R to calculate the Local Moran’s I score for each of the fibroblast subtypes of interest (46). In brief, we employed the k-nearest neighbor (k-NN) approach, using k=10 to determine neighboring cells and generate a spatial weights matrix. For each unique cell type, binary presence-absence encoding was applied, which allowed for Moran’s I calculations using the `localmoran` function for the spatial weights’ matrix. The significance of observed spatial patterns was evaluated, with Moran’s I statistics and associated p-values recorded for each sample and cell type combination. To assess the spatial organization of fibroblast subtypes, we plotted local Moran’s I score for each fibroblast subtype using `ggplot2`.

Our analysis specifically focused on cells annotated as positive for the fibroblast subtype of interest, rather than considering the entire cell population. This approach enabled a more precise evaluation of the spatial patterns unique to each fibroblast subtype. Additionally, it facilitated clearer comparisons of Moran’s I scores between NH and RA samples, highlighting potential differences in fibroblast organization between these conditions. The distribution of the local Moran’s I scores for each of the fibroblast cell types was plotted using both a gaussian distribution function with `dnorm` and raw histogram (bin size = 30) using the ggplot2 package. The mean of the gaussian distribution was calculated and plotted as single vertical line on the plot.

#### 2.4.5 Xenium pathway analysis

Differential gene expression analysis between RA and NH samples was run for each of the nine fibroblast subtypes, as defined by reference-based annotation, using the FindMarkers function in Seurat. The cut-off for significantly differentially upregulated genes was set as log2 fold change of 0.58 and FDR <= 0.05. Clusterprofiler (47) packages were used on Gene Ontology Biological Processes (GO BP) for pathway analysis. The package applies the hypergeometric test to compute p-values, which indicate the likelihood of finding a particular number of enriched genes in a gene set, as compared to what would be expected by random chance from the background gene distribution. Then adjusted p-values are calculated by applying a Benjamini-Hochberg (BH) correction. The plots were created using ggplot2 (48) and enrichplot (49) packages in R.

## 3 Results

### 3.1 Multimodal spatial omics pipeline design, quality control, validation, and benchmarking

#### 3.1.1 Ensuring a homogenous, high quality and well-annotated sample set prior to undergoing spatial omic technology evaluation

When adopting spatial biology technologies, it is critical to ensure the quality of all histological processes; from ensuring high quality of the FFPE samples upfront to maintaining excellent histological rigor when building serial sets of multi-tissue unstained slides (**Figure 1**). For this current study, 12 large synovial specimens were included based on optimal tissue architecture and the presence of synovial lining (SL) and synovial sublining (SSL) (**Figure 1A**, **Supplementary Figure 1A**). Following histopathological examination by a trained pathologist, all RA samples were classified as lympho-myeloid ‘pathotype’ except for RA2, which showed a single small focus of B-cells and was classified as the diffuse myeloid ‘pathotype’. Additional quality assessments of the samples included immunohistochemistry to confirm disease pathotypes as well as the spatial localization of canonical markers of immune cells and fibroblasts in the RA synovium compared to healthy synovium. For all RA samples, SL contains CD68^+^ macrophages and CD248^+^ PDPN^+^ PDGFRβ^neg^ and CD90^neg^ fibroblasts. Two distinct sublining histological regions of the RA synovium were noted; a superficial immune-rich SSL area (immune SSL) and a deeper fibrous area of the SSL (fibrous SSL that are both separated by a superficial plexus of blood vessels. The immune SSL contained various counts of CD20^+^, CD3^+^, CD138^+^, and CD68^+^ cells. After confirming quality, sufficient sample size and cellular composition, ROIs from 12 synovial tissue samples were distributed in 4 glass slides, and 10 serial replicates from each glass slide were generated to be used for spatial omics (**Figure 1C**).

#### 3.1.2 Internal quality control upon completion of each spatial transcriptomics modality to ensure image-based data quality prior to downstream computational analyses

Each spatial transcriptomics assay (CosMx, Xenium, and Visium) evaluates the quality and quantity of transcripts that can be detected per cell, per area, or per spot. Cut-offs for the CosMx and Xenium assays were 1) a median of ≥15 transcripts per cell, 2) ≥70% of all gene transcripts on the gene panel decoded with high quality and 3) ≤5% erroneous decoding of the estimated rate of false positives among high quality transcripts. All Xenium-stained samples with the MultiTissue-Custom gene panel (including a total of 417 genes) or the 5K-gene panel (including a total of 1, 021 genes) passed the QC. One sample (RA5) failed in CosMx-1K due to tissue section lifting during the staining. For the Visium assay, the cut off required spots to have a unique molecular identifier (UMI) count of 500 or higher, which excluded four samples (RA1, RA2, NH1 and NH2) due to low UMIs detected per spot. Illustrated summaries of the final set of samples run in each platform and the resulting set for downstream analyses are provided in **Supplementary Figure 1B**.

#### 3.1.3 Validation of cell type annotations across scST platforms and disease tissue

To reveal the spatial cellular composition of the healthy and diseased synovium, the Xenium platform served as an anchor for this analysis given its single-cell resolution. A total of 989K cells from Xenium were annotated into 11 broad cell populations as well as into 42 extended cell subtypes according to referenced publicly available by the AMP2 synovial RNAseq (10). Pathologist-based visual inspection of spatial omics samples confirmed that AMP2-based annotated broad cell types (T cells, B-cells, plasma cells, macrophages and fibroblasts) mapped accurately with both Xenium 5K genes *CD3, CD8, CD20, CD68* and *CD248* and with corresponding mIF-IHC protein markers run in the same tissue section. **Figure 2A** illustrates the arrangement of cells in the RA lympho-myeloid synovium by means of IHC protein, Xenium transcripts and scRNA seq AMP Phase 2 cell type annotations; confirming CD248^+^ fibroblasts and CD68^+^ macrophages in the SL and SSL with multifocal small to large aggregates composed of a core of CD20+ B-cells surrounded by moderate numbers of CD3^+^ T cells, all enclosed by a continuous segment composed of CD138^+^ plasma cells. As per cell type annotations using the Xenium MultiTissue (**Figure 2B**), the healthy synovium has a distinct, single-cell thick, lining fibroblast layer, and a sublining composed of large numbers of fibroblasts, prominent vascular profiles, and macrophages. In contrast, the RA lining composition has a thickened layer of SL fibroblast and macrophages, a superficial immune SSL containing aggregates of B- and T cells surrounded by plasma cells with variable in size vascular profiles and a deeper fibrous SSL rich in fibroblasts and macrophages. This contrasts with health where there is no clear compartmentalization into superficial or deep sublining as well as minimal numbers of Tcells, B cells, and plasma cells.

Quantification of this cellular heterogeneity between healthy and RA synovium was then assessed and is represented with three methods: UMAPs figures, cell type proportions and absolute cell counts (**Figures 2C, D**). Using cell type annotations, UMAP representations of the 3 healthy and 6 RA synovia were built using Single R-based annotation for the MultiTissue-Custom panel. Overall, RA synovium showed an expansion of the cell types present in the healthy synovium, which includes fibroblasts, macrophages, and endothelial cells, as well as the emergence of new cell populations such as plasma cells, T cells, and B cells. Average quantitative data on cell type proportions confirmed that the healthy synovium is composed of fibroblasts (11.3% SL and 48.4% SSL) followed by vascular-related cells (14.7% endothelial cells and 13.3% mural cells) and macrophages as the primary immune cell type (9.6%). In contrast, the lymphoid-myeloid RA synovium showed immune cell infiltrates accounting for ~54% of all the cells; specifically, 22% of macrophages, 14% of plasma cells, 9% of CD4 T cells, 6% of B cells, and 4% of CD8 T cells (**Figure 2D**). Normalized total cell counts using the broad cell type annotations confirmed a 2-fold increase in lining and sublining fibroblasts, a 2.5-fold increase in endothelial cells, and an 11-fold increase in macrophages in the RA synovium (**Figure 2D**).

#### 3.1.4 Comparison across various spatial transcriptomic platforms

Image panels of cell types detected via CosMx_1K and Xenium MultiTissue-Custom platforms (top panel) and by Visium (low panel) in serial sections of an RA sample are illustrated in **Supplementary Figure 2A**. Qualitatively, each scST approach confirmed the lining and sublining organization of fibroblasts and macrophages with a lymphoid core of B cells mixed with CD4+ and CD8+ T cells, all surrounded by a large pool of plasma cells (**Supplementary Figure 2A**). Note the nuclear versus cellular boundary segmentation algorithm used in CosMx versus Xenium MultiTissue-Custom, respectively. Next, we assessed the concordance between the mini-bulk spatial transcriptomics-Visium platform and the scST platforms Xenium 5K-genes and CosMx 1k-genes, by determining the proportions of 11 broad cell types in synovial ROIs across NH (n=1), RA (n=4) and OA (n=3) samples. Quantitative comparisons were performed using Inituype-based annotations for CosMx 1K (following the vendor’s method of choice), Single R-based annotation for Xenium 5K, and to RCTD-based (a robust, cell type decomposition for Visium) (**Figure 3A**). All raw data are included in **Supplementary Table 1**. For most cell type quantification (**Figure 3A**), only minimal differences in cell composition were observed between CosMx, Xenium, or Visium. Most comparisons were not significant by the Wilcoxon test, after adjusting for multiple hypotheses. When comparing the two scST platforms, some notable exceptions included consistently higher proportions of *CD4+* T cells and lining fibroblasts in RA from Xenium and higher proportions of *CD8+* T cells in the healthy synovium from CosMx (**Figure 3A**). Some of these discrepancies in cell type proportions or annotations between scST platforms are likely due to the differences in their targeted gene panels, their cell segmentation algorithms and, in the case of NH synovium, the limited number of cells that are present in the tissue. Correlations of the major cell type proportions as identified in ROIs with Xenium 5K and CosMx-1K, as well as those predicted by RCTD with Visium show high concordance, Pearson rho 0.89-0.92 (**Supplementary Figure 2B****;** **Supplementary Table 2**). To illustrate the heterogeneity and overall cellular composition of the healthy and RA synovium, compared to the OA synovium, UMAPs were built for CosMx_1k FOVs (**Supplementary Figure 2C**). They confirmed near absence of lymphocytes and plasma cells in NH but presence of macrophages and mural cells and large numbers of lining and sublining fibroblasts. Consistent with the profound immune cell infiltration and fibroblast expansion known to accompany RA and OA (10, 50, 51), an ~8-fold and ~15-fold increase in immune and fibroblast cellularity was detected in OA and RA tissues, respectively, when compared to the healthy synovium (see raw data in **Supplementary Table 1**). Thus, the cell type annotations and associated cellular composition assessments from mini-bulk ST and scST data gathered from NH and RA samples via CosMx or via Xenium were consistent.

A final comparison of the three transcriptomic technologies aimed to evaluate their ability to identify the relative expression of selected fibroblast genes. From among the genes present in all three platforms, we took the top up- and down-regulated genes comparing i) diseased vs. healthy tissue (**Figure 3B**) and ii) SL and SSL fibroblast regardless of disease status (**Figure 3C**). RCTD of Visium spots with SL or SSL fibroblast proportion of 0.2 or greater, whose location was verified histologically, were used for the analysis. For fibroblasts, genes differentially expressed between healthy and RA tissue included *SPARC, COL1A1, PLIN2, CDH11*, and *MMP3.* These results were consistent across the three spatial transcriptomic modalities, even though the analyzed tissue area, spots, or FOV differed between technologies (*see* **Supplementary Materials, Methods**; **Supplementary Figure 1B**). For differentially expressed genes by any two technologies, we calculated the Pearson correlation coefficient based on log2 fold change between disease and healthy samples. In all three pairwise comparisons, we observed a robust correlation of 0.75 or higher (**Supplementary Figure 2D**). Heat map visualization of the Xenium MT-Custom samples for fibroblast selected genes, using the cloud-based bioinformatic platform tool BioTuring^©^, confirmed that lining fibroblasts were clearly and consistently present in the lining layer of the NH and RA synovium while in sublining fibroblasts were appropriately located deeper within the synovial tissue (**Figure 4A**). Genes expressed in lining (*PRG4, FN1, PDPN, PLIN2, MMP3*), sublining (*PDGFRB, CD34, ASPN, COMP, PI16*) and in both lining and sublining fibroblasts (*SPARC, COL1A1, CXCL12, THY1, CHD11*).

**Figure 4:**
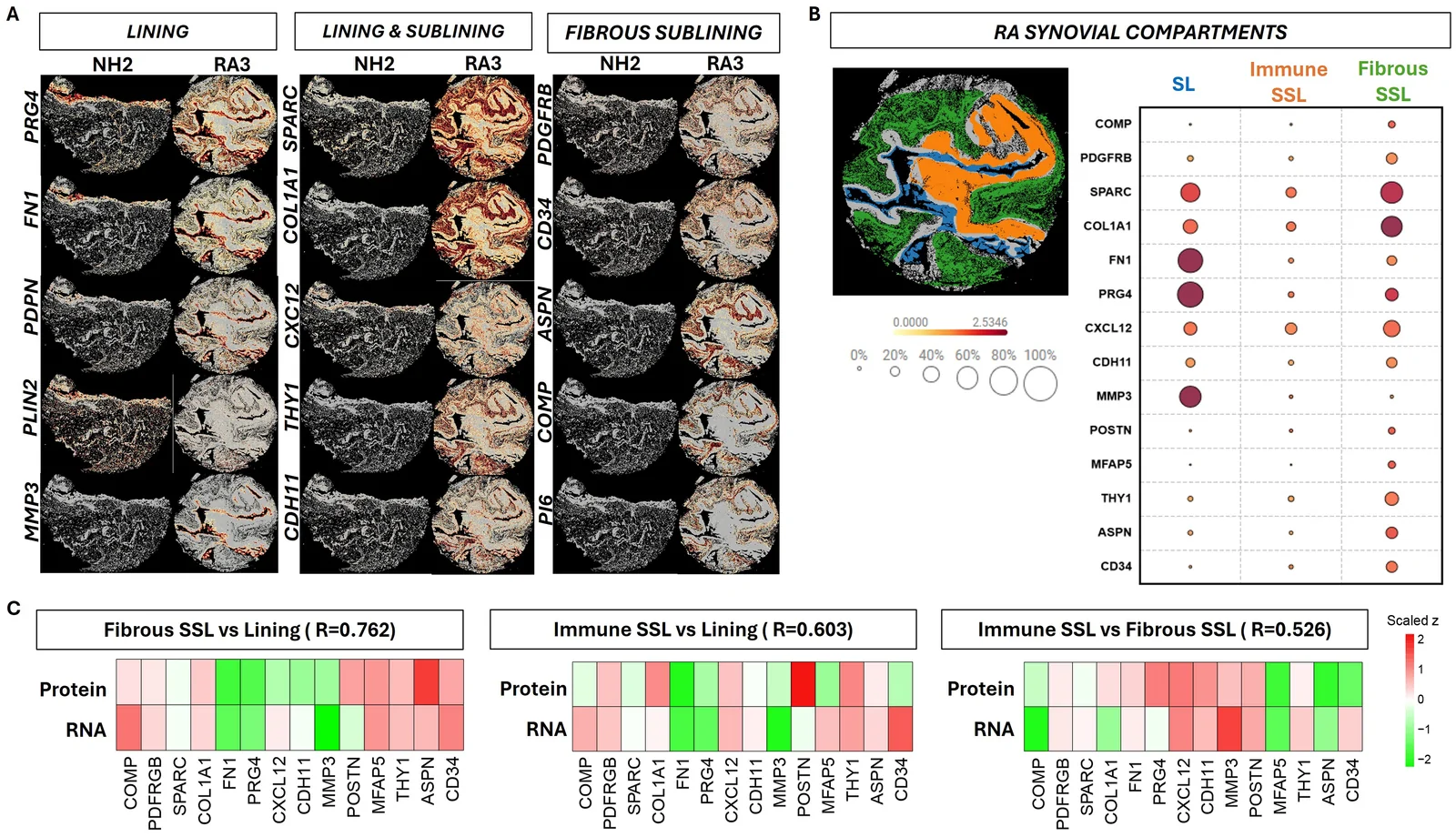
Spatial multiomics comparing single-cell spatial Xenium transcriptomics and spatial LCM-proteomics **(A)** To define fibroblast localization within the different synovial compartments, spatial heatmaps of fibroblast gene expression of select markers from Xenium MultiTissue-Custom in the NH2 and RA3 sample synovia using the Bioturing Single Cell Lens Module. **(B)** Bubble plot of fibroblast marker genes across the three synovial compartments in RA samples. Synovial compartments matching those annotated for the LCM spatial proteomics are captured as ROIs in each Xenium sample using the lasso tool in Bioturing. These analogous ROIs include synovial lining (SL), and immune and fibrous synovial sublining (SSL) as illustrated for RA3 as an example. **(C)** Pairwise differential expression analysis of RNA and protein with 14 fibroblast markers in RA fibrous SSL versus RA SL, RA immune SSL versus RA SL and RA immune SSL versus RA fibrous SSL. Pearson correlations are listed next to the comparison’s title. Pearson correlations are listed next to the comparison’s title. Log2 fold changes are scaled in each row, either Protein or RNA. The significance of correlations between 14 features with RNA vs Protein using 20, 000 permutations and a two-sided test are 0.00150, 0.0214, and 0.0532.

#### 3.1.5 Integration of Xenium spatial transcriptomics and spatial LCM LC/MS proteomics displays gene-to-protein comparison

To further extend comparisons of Xenium to proteomics-based spatial analysis, we performed LCM-proteomics on serial FFPE sections from all NH and RA samples (35). We selected ROI in H&E-stained tissue sections and then mapped them on serial sections that underwent spatial transcriptomics or spatial proteomics. For this LCM experiment, ROIs included RA SL, immune SSL, and fibrous SSL are outlined for each sample (**Supplementary Figure 1**). Pairwise comparisons of differential protein and mRNA expression for 14 overlapping fibroblast targets in those ROIs (**Figures 4B, C****;** **Supplementary Figures 3A–C**) displayed correlation coefficients with significant p-values being highest when comparing fibroblasts of the lining with the sublining (R = 0.762; p-value=0.001), intermediate for the lining vs the immune SSL (R = 0.603; p-value=0.02), and lowest for those fibroblasts of the immune SSL vs fibrous SSL (R = 0.526; p-value=0.05). Moreover, seven pairwise region expression log2 fold change comparisons (4 intra-disease-state comparisons and 3 cross-disease-state comparisons) are highlighted in **Supplementary Figure 4A**. There appeared a reasonable concordance in differential expression of genes and proteins between the different regions by scatter plots, especially in the 4 intra-disease-state comparisons as indicated by Pearson correlation (**Supplementary Figures 4B–H**). When comparing the differential expression of RA fibrous SSL area with RA SL or immune SSL areas by heatmap, fibrous SSL overexpressed *COL1A1, COMP, MFAP5, PDGFRB, POSTN* and *THY-1* while SL overexpressed *FN1* and *PRG4* at both the mRNA and protein levels (**Figures 4B, C**). Additionally, some matrisome or extracellular matrix (ECM) proteins like COL1A1, COMP, and POSTN are highly enriched in fibrous SSL areas. Secreted ECM proteins can localize quite distinctly from the mRNA restricted to fibroblast with short cytoplasmic processes and may explain discrepancies in spatial RNA and protein expression. Taken together, these data suggested the presence of distinct regional positioning of different fibroblast subtypes and prompted further investigation of fibroblast subtypes localization within healthy and RA synovial tissue.

### 3.2 Defining fibroblast subtype associations, histological patterns, spatial niches and function in RA vs healthy synovial tissue

#### 3.2.1 Multiple independent fibroblast subtypes segregate into distinct layers within the synovium

Fibroblasts are parenchymal cells of the synovium which undergo marked expansion in RA patients and contribute to pathogenesis. Given the large presence of fibroblasts throughout the synovial tissue and prior data suggesting that different fibroblast subtypes reside in different locations within the synovium, we sought to further define the topographical histological distribution of synovial fibroblast subtypes in health and RA, highlighting their spatial organization and associations with other cell types, ultimately inferring their functional role in the pathogenesis of lymphomyeloid RA.

Single-cell RNA sequencing has revealed that synovial fibroblasts segregate into distinct subsets beyond lining and sublining populations based on their gene expression (7, 10, 50–54). Thus, using the scST analysis of healthy and RA synovium with Xenium MultiTissue-Custom and 5K-gene panels we sought to better understand the changes in different subtypes of fibroblasts, including organization, expansion, spatial localization, niche or microdomain embeddings, associations with other fibroblasts or infiltrating immune cells and potential functional roles in disease. The OA samples were excluded from these deeper analyses because of their small numbers and because they were histopathologically heterogeneous.

To detect the individual fibroblast cellular subpopulations using the MultiTissue-Custom gene panel, additional annotations from the AMP2 dataset (10) were extended to include lining fibroblasts, mural cells and seven subtypes of distinct sublining fibroblast clusters, i.e., *POSTN, DKK3, CD74, NOTCH3, CXCL12, RSPO3*, and *CD34*. The five top genes used to identify each subtype are listed in **Supplementary Table 3**. Mapping of all 9fibroblast subtype annotations demonstrated the increase in fibroblast numbers in the RA synovium compared to the healthy state (**Figure 5A**). To understand the changes in the synovial composition based on fibroblast subtypes during RA disease, each fibroblast subtype was compared between the three healthy and six RA synovial samples (**Figure 5B**). We observed that proportions of lining fibroblasts, mural cells and *NOTCH3+* vascular-associated fibroblasts were all present and similarly localized in both healthy and RA synovium (18.4% vs. 14%; 17.4% vs. 13%; 6.3 vs. 6.8%, respectively). Given the vasculature expansion in RA, there were greater numbers of *NOTCH3+* fibroblasts in RA on average. Immune cell-associating *CD74+* fibroblasts were proportionally much more prevalent in RA as compared to the healthy synovium (average 22% vs. 3.4%), due to their spatial association with immune cells that infiltrated the disease synovium. Similarly, the proportion of *POSTN+* sublining fibroblasts increased in RA synovium as compared to the NH sample (average 18.5% vs. 5.1%), due to their spatial association with stromal cell proliferation, thus suggesting they may play a key role in fibroblast mediated pathogenesis. In contrast, *CXCL12+* RA fibroblasts were reduced in number and restricted to the immune-rich layer of the synovium compared to the *CXCL12*+ healthy counterpart (9.4% v. 22%, respectively), suggesting a key role in tissue homeostasis and its presence being independent of inflammation. Healthy *CD34+* fibroblasts were more predominant than in the RA synovium (13.3 vs. 3.4%). In healthy, *CD34+* and *CXCL12+* fibroblasts were scattered within the sublining of healthy synovium compared to more restricted spatial domains in the RA deep sublining and immune layers, respectively, in RA synovium. Similarly, *RSPO3+* fibroblasts, despite having similar proportions in the NH and RA synovium (11% vs. 10%), were scattered deep within the sublining in healthy synovium, which contrasts with this fibroblast subtypes being strongly associated with the lining layer in diseased synovium (**Figure 5C**).

**Figure 5:**
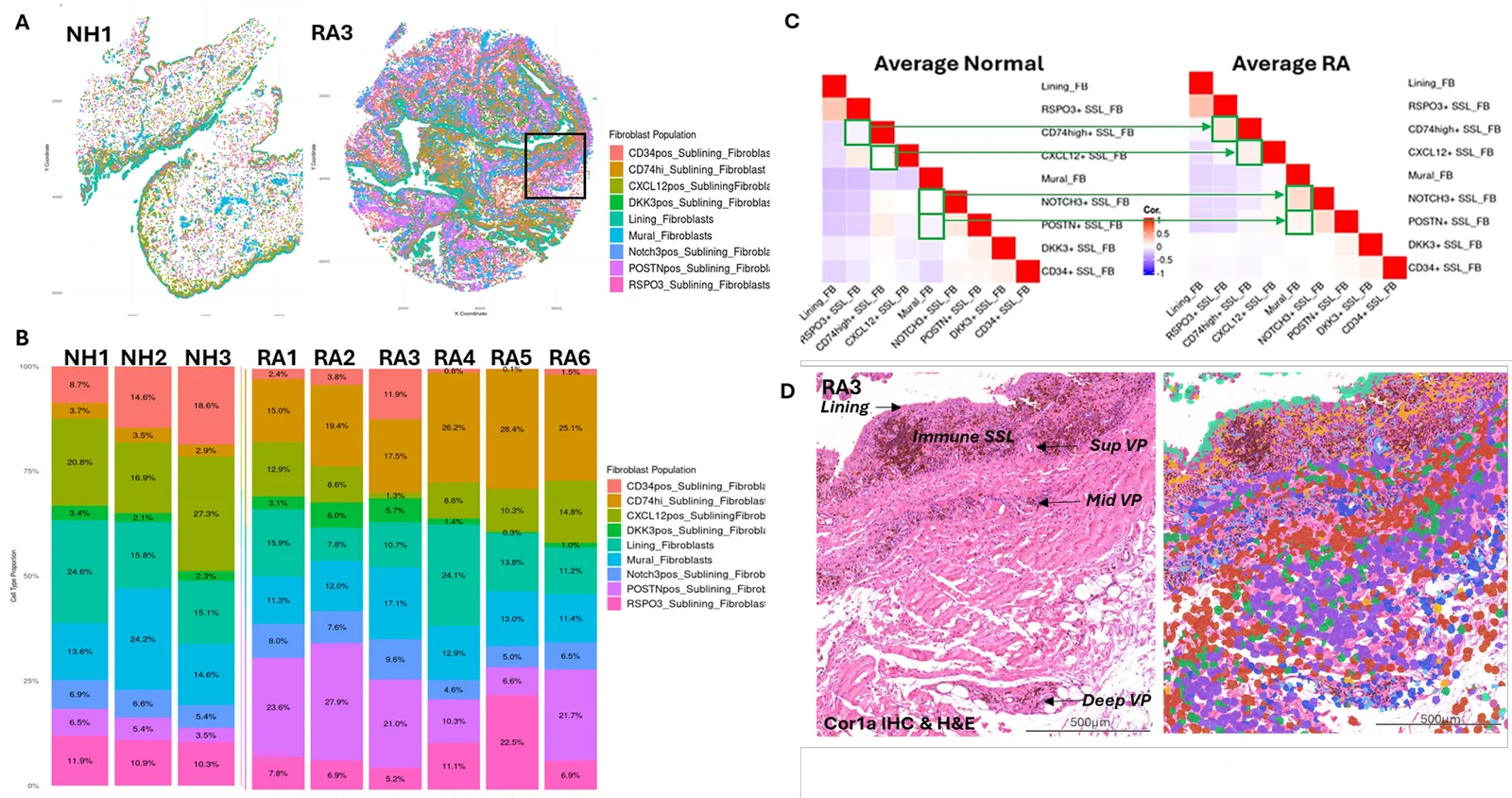
Single-cell spatial transcriptomics reveal the spatial distribution of distinct fibroblast subtypes and unique spatial neighborhood interactions. **(A)** Representative spatial plots displaying fibroblast subtypes in the representative healthy (NH1) and RA (RA3) disease sample. **(B)** Stacked bar plots of fibroblast subtype proportions (relative to total fibroblast cell count) for all healthy (NH1-NH3) and RA (RA1-RA6) samples. **(C)** Heatmaps of average Pearson correlation scores between fibroblast neighborhoods for healthyand RA synovium. The magnitude of the correlation coefficient corresponds to the color scale of the heatmap, ranging from blue (-1) to white (0) to red (1). Major changes in interactions between RA fibroblasts compared to healthy fibroblasts are highlighted in green. **(D)** Dual chromogenic immunohistochemistry for coronin 1a counterstained with an H&E image of the representative RA3 sample with the overlay of fibroblast subtypes colored according to the fibroblast subtype key in A (using Xenium MultiTissue_Custom gene panel). The superficial, mid, and deep vascular plexi (VP) are highlighted on the H&E.

A further effort to objectively assess the distinct multilayer pattern of fibroblast subtypes across all RA synovial samples focused on the computational evaluation of spatial neighborhoods of the different fibroblast’s subtypes included (**Figure 5C**, **Supplementary Figure 5**) in all RA and NH samples. The hoodscanR package was used to determine correlation between the identified cellular neighborhoods for each of the fibroblast subtypes. This analysis confirmed changes in cell-to-cell proximities by displaying a heatmap of the Pearson correlation coefficients between neighborhoods, with an increase in fibroblast subtype interactions in RA compared to the NH synovium (**Figure 5C**). In particular, the following interactions exhibited significant greater correlation in RA: *RSPO3+* and *CD74+* fibroblasts, *CD74+* and *CXCL12+* fibroblasts, and mural fibroblasts with *NOTCH3+* fibroblasts and with *POSTN+ fibroblasts*. These results indicate that in RA, *CD74+* fibroblasts subtypes become strongly associated with *RSPO3+* fibroblasts and less associated with deeper sublining *NOTCH3+*, *DKK3+*, *POSTN+* and *CD34+* fibroblasts (**Figure 5D**). Additionally, the vascular stroma appeared more organized in RA than in NH, with mural fibroblasts, *NOTCH3*+ and *POSTN+* fibroblasts displaying higher correlation scores. **Supplementary Figure 5C** discloses the Pearson correlation neighborhood for all examined samples. Thus, the neighborhood profiles sho increased spatial proximities between *CD74+*, *RSPO3+*, *NOTCH3*+, and mural fibroblasts in RA but not in NH.

Notably, the pathologist-based evaluation of a combined IHC for coronin-1a (which detects leukocytes) and H&E-stained tissue sections overlaid on the Xenium fibroblast subtypes, revealed the spatial laminar distribution and distinct fibroblast arrangement within the sublining of the RA synovium (**Figure 5D**). Specifically, directly beneath the lining layer was a superficial sublining layer composed of the RSPO3+ fibroblast subtype and a few mural cells. Beneath that layer and interspersed with the coronin-1a^+^ immune cell aggregates and superficial vascular plexus, abundant*CD74+*, mural, and, to a lesser extent, *CXCL12+* fibroblast subtypes. The *NOTCH3+* fibroblast subtype was strongly associated with the vasculature and mural fibroblasts, consistent with these cells responding to NOTCH3 signaling from endothelial cells (55). In the stromal rich sublining, layers enriched in*POSTN+, DKK3+* and *CD34+* fibroblast subtypes were not associated with either vasculature or immune aggregates but rather interwoven within bands of connective tissue as identified on H&E stain (**Figure 5D**). The *POSTN+* fibroblast subtype, a variably thick band that mostly overlapped with the *DKK3+* subtype, runs dorsally and ventrally to CD34+ fibroblasts along the mid vascular plexus with a few extended processes nearing mural and *NOTCH3+* fibroblasts. In deeper ventral areas with less tightly packed connective tissue, *POSTN*+, *DKK3*, and *CD34+* fibroblasts subtypes were overrepresented. This laminar organization was particularly obvious in the RA3 donor whose synovial section orientation was longitudinal and allowed for full thickness evaluation of all layers (**Figures 5A, D**).

Collectively we find i) the expansion of CD74+ and POSTN+ fibroblasts in the immune and stromal synovial compartments, respectively, ii) the redistribution or differentiation of CXCL12+, CD34+ and RSPO3+ fibroblasts within restricted spatial domains in RA, iii) and the formation of closer neighborhood associations between *CD74+*, *RSPO3+*, *NOTCH3+* and mural fibroblasts and highlight the stromal changes that occur in RA synovium as compared to its healthy milieu (**Figure 5**).

#### 3.2.2 Fibroblast subtypes are localized in cellular niches reflective of function and pathophysiology in diseased tissue

To further investigate cellular associations with other cell types, niches, or spatial arrangements of fibroblast subtypes regardless of the orientation of the tissue section, we determined distances of fibroblast subtype clusters to landmarks within the tissues. We focused on understanding the multicellular niches in relation to infiltrating B cell rich aggregates (B cell niche) in RA synovium as well as around sub-lining vasculature (vascular niche) in both NH and RA synovium (**Figure 6**). For microdomain characterization, we identified B cell niches using an image analysis thresholding algorithm in Visiopharm to identify *CD20+* hotspots (i.e lymphoid aggregate clusters on the H&E image) from the mIF image, and vascular niches using pathologist annotations on H&E-stained sections. Once these ROIs were identified, they were expanded in intervals of 15µm up to 75 µm to effectively capture the central and surrounding compartments within each niche category. Following this process for ROI generation, we integrated AMP-II RA based fibroblast subtypes within each B cell and vascular niche, respectively (**Figures 6C, D**). For the B cell niche, these analyses identified consistent cellular composition of the aggregate core, intermediate, and distal perifollicular microdomains in all RA samples. Accordingly, microdomains within 30µm from the core of B cell aggregates revealed greater abundance of *CD74+* SSL fibroblast and slightly greater abundance of mural fibroblasts (**Figure 6C**) with peak distances of 15um from the center of the cluster. As expected, lining fibroblasts appeared in a gradient fashion starting from 15µm away from the B cell cluster and peaking at 75um distance, reflecting the location of B cell aggregates in the superficial sublining. Collectively, these results demonstrate a unique tri-fold compartmentalization of fibroblast subtypes around these lymphoid aggregates, with *CD74+* cells at the central core of the aggregate, mural fibroblasts potentially as an indication of concomitant capillaries, and then by lining fibroblasts that overlay the immune aggregate layer.

**Figure 6:**
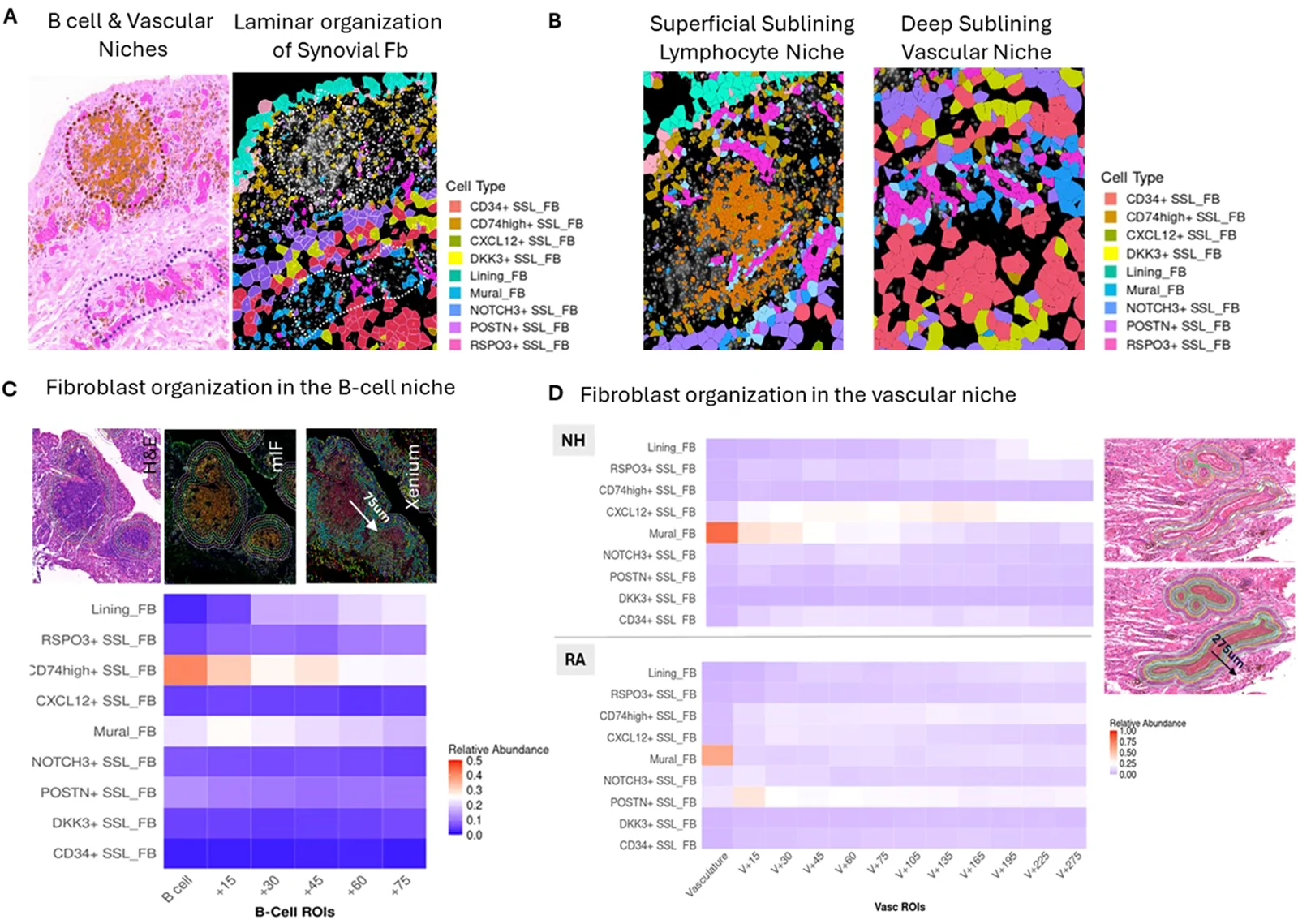
Expansion and spatial organization of fibroblast subtypes in RA synovium, and their lymphoid, vascular and ECM enriched microdomains. **(A, B)** Overlay of fibroblast subtypes on an H&E image, with dotted ROIs corresponding to the B Cell **(A)** and vascular **(B)** niches. C) Heatmap of relative abundance of fibroblast subtypes within the B Cell niche, with the magnitude of abundance corresponding to the color scale range from blue (0) to white (0.5) to red (1). **(C)** Panel of post-Xenium 5K panel H&E image with identified B cell niche core (center circle) and expanded sub-compartments (15µm increments, max 75µm), paired with post-Xenium 5K panel multiplex IF-IHC (mIF) image (orange corresponding to CD20+ signal) and overlaid annotated fibroblast subtypes. **(D)** Heatmaps of relative abundance of fibroblast subtypes within the Vascular niches of healthy (NH) and RA, with the magnitude of abundance corresponding to the color scale range from blue (0) to white (0.5) to red (1). H&E images with pathology annotated vasculature, with circular grids corresponding the expanded ROI sub-compartments (15-30µm increments, max 275µm).

For the vascular niche, including the immediate perivascular stroma, we found that while an expected abundance of mural fibroblast subtypes occurs in the central core of the vasculature for both RA and NH samples, these cells are more restricted to the core of the vasculature in RA, with an increased presence of *NOTCH3+* and *POSTN+* sublining fibroblasts associated with the vasculature (**Figure 6D**). Additionally, the localization of *CXCL12+* fibroblasts distal to the vascular core in NH samples is, in fact, reduced or eliminated in RA samples, indicating an altered localization of this fibroblast subtype relative to vessels in disease.

#### 3.2.3 Increased spatial organization of fibroblast subtypes in diseased tissue

To further quantify the degree of cellular organization and compare spatial organizational levels of the different fibroblast subtypes between healthy and RA synovium we computed the spatial autocorrelation of individual fibroblast subtypes using the local Moran’s I score. This clustering score assesses the spatial relationship of each cell type and ranges from -1 (dispersed) to 1 (clustered) with a value of 0, or close to 0, when it is randomly distributed. First, we computed the local Moran’s I score for each of the fibroblast subtypes within the NH1 and RA3 samples for preliminary analysis and plotted the scores in cartesian coordinate space (**Figure 7A**). Visual and computational assessments of the score patterns in each of the sample groups revealed markedly higher clustering scores in the RA synovium than NH for the majority of fibroblast subtypes, with major differences observed in mean Moran’s I for lining (106.73 in RA vs. 22.06 in NH), *RSPO3+* (64.12 in RA vs. 9.75 in NH), and *CD74+* (69.43 in RA vs. 14.74 in NH) fibroblast subtypes (**Figure 7B**). We found significantly larger mean local Moran’s I autocorrelation scores for all fibroblast subtypes in RA vs. NH (e.g., greatest increase in *CXCL12+* vs. *CD34+* fibroblast organization) (**Figure 7C**). Thus, these data confirm an increase in organizational status with less random distribution of several RA fibroblast subtypes compared to healthy synovium.

**Figure 7:**
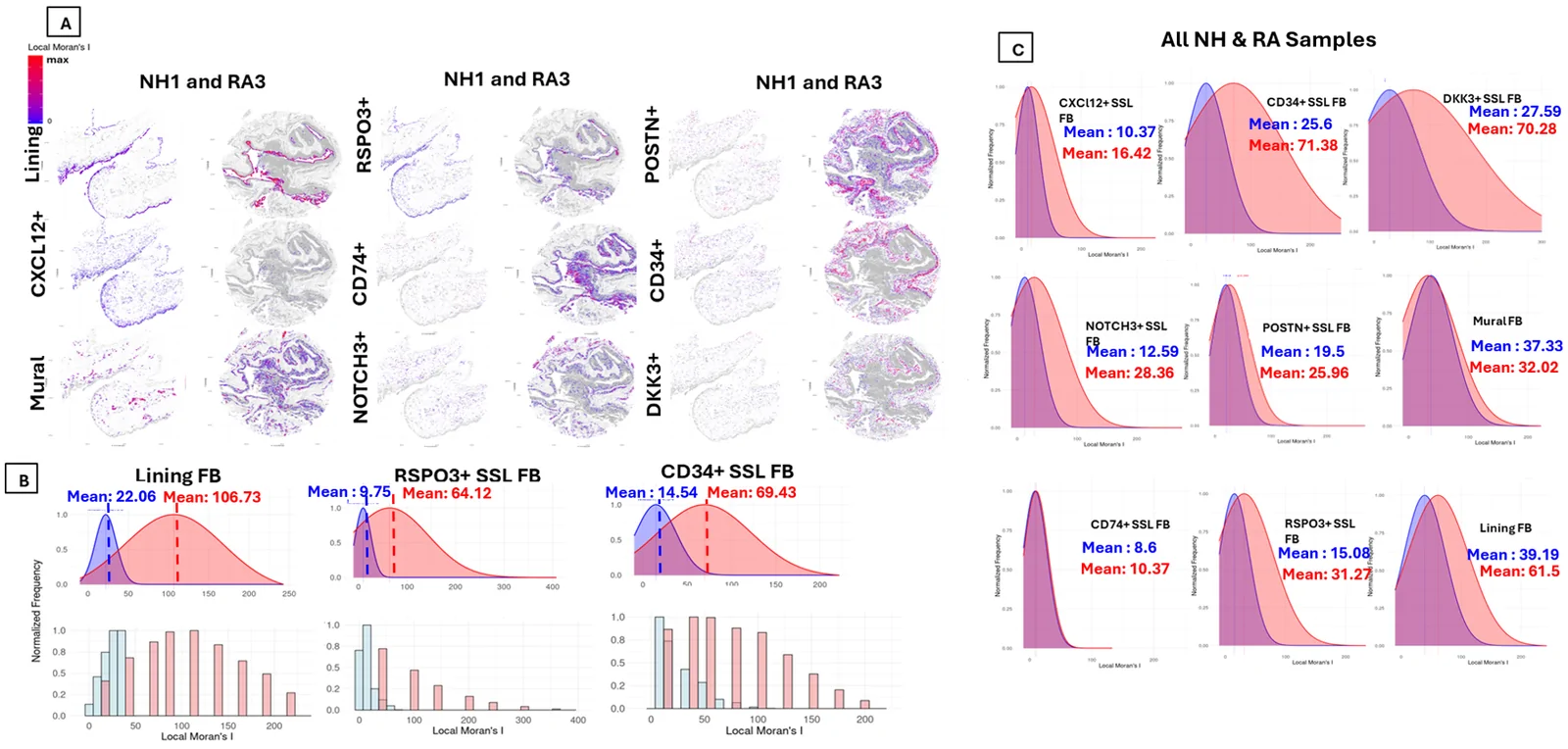
Fibroblast subtypes demonstrate increased spatial organization and a novel ‘*de novo*’ layering pattern in the RA diseased synovium. **(A)** Spatial plots of the local Moran’s I scores for each of the fibroblast subtypes in the representative RA (RA3, left columns) and healthy (NH1, right columns) samples. The range of scores is displayed on a blue-red-colored scale (*e.g.*, ranging from 0, blue to max, red), with grey denoting cells in the background (outside population of interest). **(B)** Histograms and gaussian-fit curves for the local Moran’s I scores for the lining (left), RSPO3+ (mid), and CD34+ (right) fibroblast subtypes that show the greatest difference between healthy (NH1 -blue) and RA (RA3 -red) samples. **(C)** Histograms and gaussian-fit curves of the local Moran’s I scores for all fibroblast subtypes across all healthy(blue) and RA (red) samples.

#### 3.2.4 Potential functional roles of fibroblast subtypes in disease

To explore the possible functions or pathophysiology of the different fibroblast subtypes in RA as compared to the healthy synovium, we performed Gene Ontology (GO) analyses on fibroblasts subtypes annotated using the MultiTissue-Custom panel. Based on pathways altered in the different fibroblast subtypes they fell into one of four functional categories (**Figure 8**): i) pro-inflammatory (lining, *RSPO3+, CXCL12+*); ii) cell projection organization/migration (*POSTN+, CD74+*, Mural); iii) vascular-related (NOTCH3+) and iv) development and differentiation pathways (*CD34+, DKK3+*). Additionally, these functional changes were associated with localization of the fibroblast subtypes in RA. The lining, *RSPO3+* and *CXCL12+* fibroblast subtypes which show increases in proinflammatory pathways in RA synovium are located interspersed with the inflammatory aggregates. The *POSTN+*, and mural cells that show pathways related to cell projections and migration are encircling the vasculature. NOTCH3+ fibroblasts are strongly associated with vasculature, and these pathways may indicate the acquisition of certain vascular functions in diseased tissue. The *CD34+* and *DKK3+* fibroblast subtypes that show increases in pathways related to development and differentiation in RA synovium are located deep in the sublining and may serve as a reservoir for developing fibroblasts. Thus, changes in functional attributes in the disease state are associated with specific groups of fibroblast subtypes.

**Figure 8:**
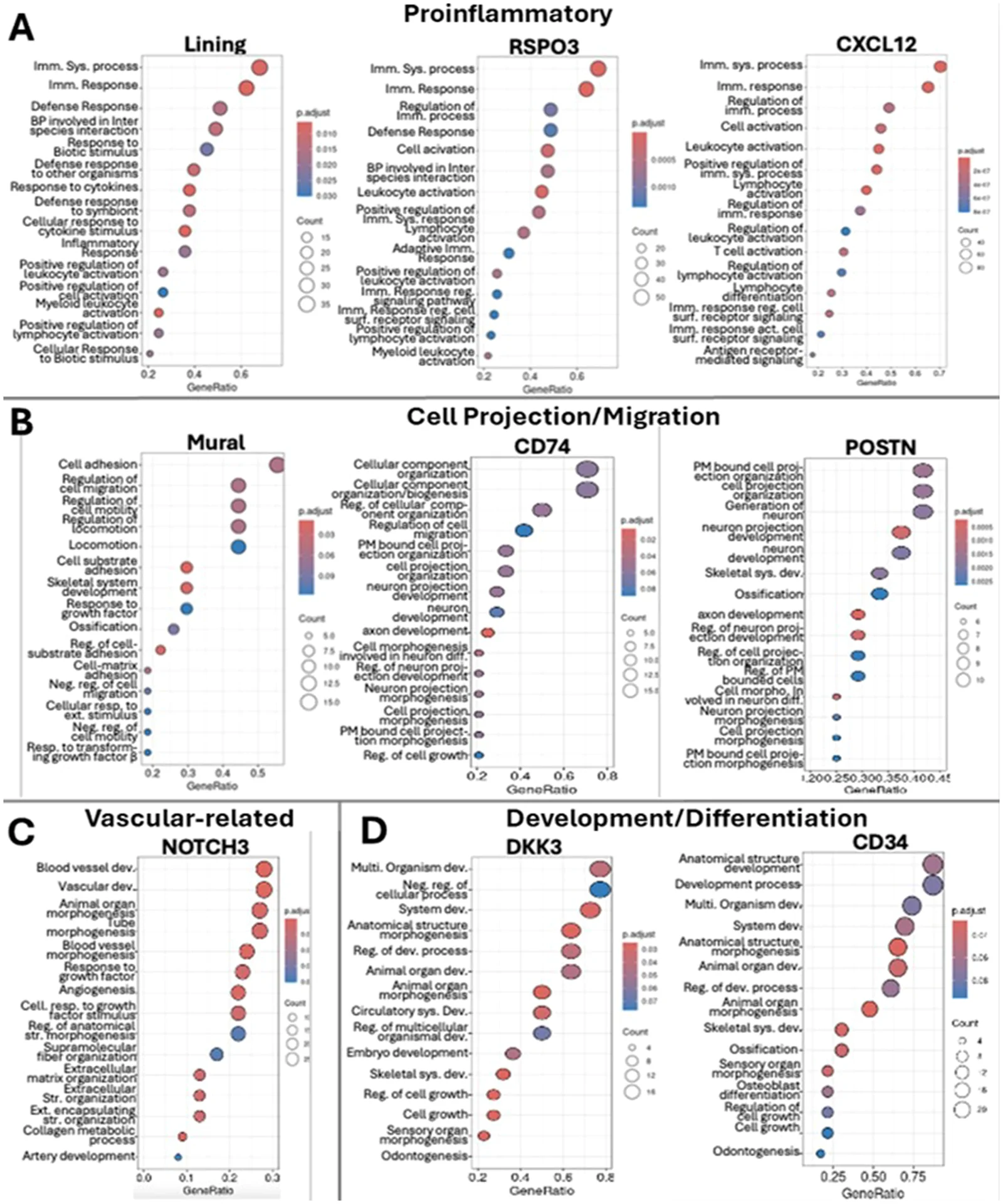
Pathway analysis of fibroblast subtypes in synovium. Dot plots show Gene Ontology Biological Processes (GO-BP) pathways upregulated in disease compared to the healthy synovium in the nine fibroblast subtypes. Pathways categorized the fibroblast subtypes as **(A)** Pro-inflammatory, **(B)** Cell Projection/Migration, **(C)** Vascular-related and **(D)** Development/Differentiation. All dot plots are from Xenium MultiTissue-Custom panel data except NOTCH3+ fibroblasts which are from Xenium 5K Panel data as no differential pathways were identified in the MultiTissue-Custom panel. The cut-off for significantly differentially upregulated genes was set as log2 fold change of 0.58 and FDR <= 0.05.

## 4 Discussion

This spatial multiomics study sought to first evaluate different spatial transcriptomics platforms in serial sections of relatively large biopsies of synovial tissue from RA, NH and OA subjects and second, to leverage the combination of single-cell spatial transcriptomics and proteomics with histology (multimodal approach) to advance our understanding of spatial localization and organizational pattern of fibroblasts, including cell-to-cell interactions, fibroblast subtypes and cellular niches in the RA synovium. Starting with histological assessment of major synovial compartments and the topography of major cell populations, the investigation moved to identifying a workflow that was able to spatially define cellular subtypes and RA niches with specific cellular interactions. This was followed by an in-depth assessment of fibroblast subtype localization in healthy and disease states. Conclusions from this work include a high concordance of local cellular composition across spatial transcriptomics platforms, and the description for the first time of the distinct laminar topography of synovial fibroblasts in the RA synovial lymphoid-myeloid sublining highlighting the enrichment of specific fibroblast subtype neighborhoods functionally reorganized in disease.

Our findings confirmed previous pseudo-bulk spatial transcriptomic studies on RA synovial tissue that discretely define relationships between cell types in the lining and in ectopic structures (24, 39). More recent studies investigating fibroblasts within the synovium of RA patients used single-cell RNA sequencing, ATAC sequencing for transcription factor profiling and Visium technologies to classified fibroblasts as activated vs resting in both the lining and sublining compartments as well as intermediate populations between these states (56). Using single-cell spatial transcriptomics in RA synovial tissue, our study confirms prior observations such as the associations of *NOTCH3+* fibroblasts with vasculature and *POSTN+* fibroblast interactions with *NOTCH3+* fibroblasts (25, 54, 55, 57, 58) by mapping *NOTCH3+* fibroblasts with mural cells and vasculature in the RA setting as well as Gene Ontogeny pathways related to vasculature. NOTCH3 expression was also reported to be elevated in RA vs OA, and NOTCH3 signaling has been suggested to drive these fibroblasts to become more pathogenic as blockade can limit experimental arthritis (55). The detection of processes of *POSTN+* fibroblast in the vicinity of *NOTCH3+* fibroblasts in RA but not in NH found in our proximal to distal vascular niche analyses could suggest a supporting role to disease-associated microvascular proliferation or to infiltration of immune cells into the tissue.

Importantly, prior work using single-cell RNA sequencing in RA synovium to define molecular subpopulations (10, 50, 51) was essential for assigning the spatial localization of these populations in RA tissue in the present studies. In RA synovium, broad cell type annotations for major immune cell subsets demonstrate the expected localization of T, B, and plasma cells to lymphoid aggregates beneath the lining layer that are absent in healthy synovium. Moreover, the most intriguing finding is that distinct fibroblast subtypes, previously identified by single-cell RNA sequencing in the RA synovial sublining, appear spatially arranged in various layers underneath the lining, around immune and vasculature and throughout the deep SSL. Overall, this result strikingly demonstrates that not only do certain subtypes of fibroblasts increase in disease, but the degree of organization into layers and cellular niches is also more pronounced.

Several specific subtypes of fibroblasts emerged from our spatial analyses as notable from multiple perspectives, for example, the *CD74+* fibroblasts, which expand in RA, demonstrated increases in pathways associated with cell projection and migration, as well as tight associations with B cell aggregates found only in the disease state. Given the close association with B cell aggregates and enriched pathways reflecting motility, *CD74+* fibroblasts may serve supporting functions for the highly organized immune cell aggregates and rich capillary network. Other subsets of fibroblasts such as lining, *RSPO3+* and *CXCL12+* fibroblasts are interspersed within the immune layer and show increased pro-inflammatory pathways in RA synovium indicating that these populations may promote or respond to the inflammation occurring within diseased tissue. Our spatial multiomics integrated data mapping *CD74+* and *CXCL12+* fibroblast subtypes in LCM-dissected CXCL12 and POSTN protein-rich RA lymphoid microenvironment supports a role in immune cell recruitment, chronic inflammation and ECM remodeling. *RSPO3+* cells also appear to be either specifically recruited to or differentiate locally via Wnt signaling within these subintimal areas as they are found scattered in healthy synovium. *CD34+* fibroblasts similarly appear to relocate or locally differentiate from a scattered distribution in healthy synovium to being concentrated in the deep sublining in disease with a higher level of organization. Consistent with CD34 as a marker of stem cells, this fibroblast subtype shows pathways related to development and differentiation and may serve as a reservoir for other fibroblast subtypes from the sequestered location within the deep sublining. Thus, our detailed single-cell spatial transcriptomics approach demonstrated which fibroblast ecotypes are embedded within specific synovial compartments (i.e. lining or sublining compartments) and which ones are within RA histopathological microenvironments (i.e. lymphoid aggregates or microvascular proliferation). Taken together, these data indicate that in the RA disease synovium, certain fibroblast subtypes expand, migrate, and/or differentiate with increased spatial organization and corresponding functions around specific cellular niches.

In addition to advancing our understanding topographical cellular interactions that emerge in RA synovial niches, the serial tissue workflow and assessment of four spatial-omic platforms in this study including pseudo-bulk and single-cell resolution RNA-based platforms (Visium, Xenium, CosMx) and protein-based platforms (LCM proteomics as well as low-plex immunofluorescent IHC), with all modalities integrated with histopathology provides a foundation for running spatial omics studies. The value of this histology-based normalization in compartments of interest is critical when integrating the scST platforms directed to mini-bulk RNA Visium or protein via LCM spatial proteomics. An additional critical step for scST is the selection of a gene panel complementary to the scRNA seq datasets used for annotating the cell types spatially. Our spatial transcriptomics work using Visium, Xenium and CosMx also emphasizes the importance of benchmarking new spatial data to existing well-established single-cell RNA seq datasets (48, 52). Our comparison of various cell type proportions in the RA and NH synovium between single-cell transcriptomics platforms confirmed an overall concordance between CosMx and Xenium technologies when serial sample sets were normalized by cell type annotations, area size and region. Additionally, general expression trends were not different when comparing expression of various fibroblast genes detected by CosMx, Xenium or Visium in healthy and RA serial sample sets, or between RA synovial lining and sublining compartments even when they differ in the size of the field of view under analysis (as detailed in **Supplementary Figure 1**). Thus, these studies advance the workflow for spatial multi-omics on the same tissue section, compare the current spatial platforms, and emphasize the importance of histological integration of data as part of the spatial omics pipeline. While comparing data from multicellular FOVs analyzed by various platforms proved successful, it is our recommendation to prioritize multimodal approaches run on the same section when single-cell resolution is a requirement, rather than in serial sections since the co-registration after aligning serial sections tends to be suboptimal at single-cell level.

Each of these technologies also has advantages depending on the biological questions. For the interrogation of fibroblast heterogeneity at a subtype level, using cell deconvolution of Visium spots was insufficient compared to Xenium. Similarly, single-cell resolution was not feasible using our LCM proteomics approach due to inaccurate manual annotation of tissue layers of fibroblasts identified by Xenium to be captured by LCM-proteomics (not shown) in an H&E-stained serial section. However, our multiomics approach provided significant biological insights. First, our Xenium scST data confirmed the presence of niches containing distinct types of fibroblasts, and their close relationship to other cell types. Second, by running Xenium and multiplex IHC on the same section, and LCM-proteomics in a serial section, we demonstrated the close spatial relationship of CD74+ sublining fibroblast (identified by scST) to the core of B cell rich areas (identified by IHC), where CXCL12, POSTN, PDGFRB and CD90 proteins are high in the lymphoid-stromal microenvironment (identified by LCM-proteomics). These combined approaches work towards technological and computational methods that will allow for scaling up to the whole transcriptome with spatially resolved cell to cell interactions, neighborhoods, and niches.

Limitations of this study include low sample number, limited patient clinical information, patient-to-patient variability and the use of previously reported gene signatures for cell populations vs defining these *de novo* from our data. While the multi-omics approach applied in the present studies was comprehensive and used large pieces of histopathologically confirmed active RA tissue, the samples were limited and had a paucity of clinical information, such as disease activity, seropositivity, disease duration, or treatment history. Given the paucity of samples for OA (3 patients) and the heterogeneity observed, we limited our deep spatial analyses to the RA samples and excluded OA; thus, it remains to be confirmed if fibroblast topography is a RA disease-specific finding or an epiphenomenon of the diseased synovium. Patient to patient variability was also observed within diseased and healthy subjects with respect to proportions of the different fibroblast subtypes. In addition to intrinsic histological variability, such differences can also arise due to the plane of sectioning, tissue orientation, or serial sectioning. For example, a longitudinal orientation will display all histological layers and cell types herein described from the inner SL to the deep fibrous SSL whereas samples with a cross-sectional orientation may display only those compartments represented at that level of depth. An example of changes in proportions of fibroblasts between serial sections of the same sample is shown in **Supplementary Figure 5**. In this respect, spatial analyses of proportions should be interpreted as a biological feature/trend, and it is insufficient for patient stratification purposes. Lastly, the use of previously reported data from single-cell RNAseq as a basis for cellular populations may have limited our ability to define new, structurally distinct populations within tissue that would not have been captured with a single-cell analysis of synovial tissue homogenate.

As spatial technologies continue to evolve to include state-of-the-art single-cell spatial transcriptomics integrating the whole transcriptome as well as cyclic multiplex IF-IHC running dozens of proteins, will allow for future application of spatial multiomics to clinical biopsies. As a gold standard practice, we have run our spatial multiomics in synovial large sections with good histology and representation of all morphological compartments of the synovium to serve as a histomolecular reference of cell subtypes, niches and neighborhoods, and guide interpretation in smaller pieces of synovium acquired as needle biopsies in clinical practice, where under-representation of specific subtypes of fibroblast might be the consequence of loss of the integral tissue microarchitecture. Application of these spatial omic methodologies with greater computational accuracy to more patients with various clinical criteria (i.e. response/non-response to therapy, pathotype, seropositive/negative) and with additional quantifiable measures will help understand how universal these observations are and/or if additional patterns emerge with different clinical or pathotype features. Equally important, if the relevance of the spatial composition of the RA synovium underscores pathotypes, endotypes or remission states, it will provide a valuable opportunity to use spatial omics in synovial biopsies to understand patient heterogeneity with the aim of further stratifying patients and identifying biomarkers and targets for patients inadequately responding to current therapies. Additionally, future studies comparing RA to other synovial diseases should help understand the relevance of fibroblasts in RA pathophysiology. It remains to be determined which, if any, of the fibroblast subtypes contribute to pathogenesis as well as whether the observed topography and heterogeneity of fibroblasts are driven by RA or is in part an epiphenomenon resulting from the injury to the synovium and repair mechanisms.

## 5 Conclusions

In summary, this work confirms prior reports and expands our view of the complex cellular histological organization within the RA compared to the healthy synovium. Through histologically resolved spatial multiomics technologies, we have revealed that fibroblast subtypes in diseased synovium exist in spatially distinct locations and form multilayered organization and structures beyond lining and sublining. These current spatial-omic analyses highlight how cellular localizations, cell-to-cell interactions, and potential cellular functions within tissue can be identified at the single-cell level. It is anticipated that this increased resolution and depth of spatial omics applied to clinically relevant samples will facilitate the development of new therapeutics for patients and biomarkers to aid in treatment decisions. This work provides a foundation for larger future studies that will provide additional insights related to patient populations, therapeutic response, and therapeutic targets.

## Data Availability

The Visium and Xenium data presented in the study are deposited in the GEO repository, with GEO IDs accession numbers GSE347797 (https://www.ncbi.nlm.nih.gov/geo/query/acc.cgi?acc=GSE347797) and GSE347958 (https://www.ncbi.nlm.nih.gov/geo/query/acc.cgi?acc=GSE347958), respectively.
