# Supplementary material for: Spatial multiomics identifies rheumatoid arthritis synovial niches and de-novo laminar histological patterns of fibroblasts subtypes
Source: Front Immunol. 2026 Oct 16;17:1883263. doi: 10.3389/fimmu.2026.1883263 (PMC13719634; doi:10.3389/fimmu.2026.1883263)
Supplement: Supplementary file 1 [file DataSheet1.docx]

**SUPPLEMENTARY MATERIAL**

**Spatial multiomics identifies rheumatoid arthritis synovial niches and de-novo laminar histological patterns of fibroblasts subtypes**

Sílvia Sisó^#1^, Archana Iyer^2^, Vineeth Raghuram^1^, Neha Chaudhary^2^, Heather Knight^1^, Fei Wang^1,*^, Ioan Filip^2^, Lori Duggan^1^, Xue Wang^3^, Katherine Naughton^3^, Terry Melim^1,*^, Anna Yarilina^1,*^, Yingli Yang^1^, Jocelyn Rivas^1^, Abby McCarthy^1^, Yu Tian^1^, Dan Chang^2,*^, Timothy R.D.J. Radstake ^2,*^ , Melanie C. Ruzek^1,*^, Annette Schwartz-Sterman^1^

^1^ Immunology Discovery Research, AbbVie Bioresearch Center, 100 Research Drive, Worcester, MA 01605, USA

^2^ Immunology Discovery Research, AbbVie, Cambridge Research Center, 200 Sidney Street Cambridge, MA 02139, USA

^3^ Immunology Discovery ResearchResearch & Development, AbbVie Inc., North Chicago, IL 60064, USA

 *Former AbbVie Employee

^#^ corresponding author: [silvia.siso@abbvie.com](mailto:silvia.siso@abbvie.com)

Content:

- Supplementary Materials & Methods:
  - Demographic subject information
  - Spatial-omics bench pipeline
  - QC of single cell spatial transdcriptomics gene panel (CosMx 1K; Xenium MT and Xenium 5K) marker gene heatmap and bubble plots of major cell type
  - References
- Supplementary Table 1
- Supplementary Table 2
- Supplementary Table 3
- Supplementary Figure 1
- Supplementary Figure 2
- Supplementary Figure 3
- Supplementary Figure 4
- Supplementary Figure 5

**Supplementary Materials & Methods**

**Demographic information of subjects on condition, sex, age, joint site and diagnosis on synovium:**

- NH1: Healthy, Caucasian, 55 years-old, M, Knee, Non-lesional
- NH2: Healthy, Caucasian, 96 years-old, F, knee, Non-lesional
- NH3: Healthy, Caucasian, 75 years-old, F, Knee, Non-lesional
- RA1: RA, Caucasian, 31 years-old, F, unknown, Lymphomyeloid
- RA2: RA, Unknown, 61 years-old, F, Hand, Diffuse Myeloid
- RA3: RA, Unknown, 75 years-old, F, Hand, Lymphomyeloid
- RA4: RA, Unknown, 68 years-old, F, Biopsy, Lymphomyeloid
- RA5: RA, Caucasian, 57 years-old, M, Knee, Lymphomyeloid
- RA6: RA, Caucasian, 60 years-old, M, Hip, Lymphomyeloid with tertiary-like structures
- OA1: OA, Unknown, 52 years, F, Knee
- OA2: OA, Unkown, 47 years, M, Knee
- OA3: OA, Unknown, 54 years, F, Knee

Some information was unavailable. Sex was not considered a biological variable. For more details on samples see Wang et al (2025).

**Spatial-omic technologies: bench work**

*Spatial Proteomics*

Using LCM-proteomics, 5 micron-thick sections were mounted on PEN Membrane slides (Leica, Cat #11505189) and stained with Meyer’s hematoxylin for 30 seconds then held at 4°C until use. A serial H&E slide (Surgipath Apex Superior adhesive slides, Leica, Cat #3800080) was digitally imaged using a Panoramic 250 whole slide scanner at 20x magnification (3DHISTECH) and imported into Visiopharm software for annotating regions of interest (ROI) by a pathologist. ROIs included SL as well as areas rich in immune cells (immune SSL) or in fibrous stroma (fibrous SSL). Such annotated H&E images were used to guide the manual LCM capture on the hematoxylin-stained PEN membrane slides. For each ROI, ~2 mm^2^ of tissue was captured into 96-well plate (Eppendorf, Cat #0030129512) and placed at -80 °C. Details on sample preparation for proteomics as well as successful protein ID yields using synovial LCM sizes of 0.5 mm^2^ are given in our previously published LCM proteomics work (33).

For spatial transcriptomics, we focused on three different platforms, the mini-bulk sequencing-based Visium, and the two single cell imaging-based Xenium and CosMx. For Xenium and CosMx, initial steps included selecting the off-the-shelf panel and customization.

*CosMx Method*

11-15 ROIs from each 5µm section of synovium were placed on a Leica glass slide and were submitted to the Nanostring state-of-the-art laboratory (Bruker, Seattle) as part of the Technology Access Program to run CosMx Spatial Molecular Imaging platform and measure expression of 1K genes including 24 custom genes.  The tissue section from RA5 was damaged during the staining process in the CosMx platform.

*Custom Genes Added to the CosMx 1K Panel*

gene

ABI3BP

ASPN

CD90 (THY-1)

CLIC5

COMP

FBN1

HBEGF

IGFBP4

LRRC15

MALT1

MMP3

MYD88

NNMT

PAPP-A

PDPN

PI16

PLIN2

POSTN

PRG4

PTPN22

RANKL

SIRPA

SPARC

TAB1

*Xenium Method*

5 µm sections of synovium were placed on the Xenium Slide following the Xenium In Situ for FFPE – Tissue Preparation Guide (CG000578) protocol (10x Genomics).  The slides were stored at room temperature in a desiccator before proceeding to decrosslinking.  Xenium In Situ for FFPE – Deparaffinization & Decrosslinking (CG000580), Xenium In Situ Gene Expression (CG000582) and Xenium Analyzer (CG000584) protocols were used to process the slides.  The slides were stained with hematoxylin and eosin on the Leica ST5010 auto-stainer using the same reagents and timing found in the Xenium In Situ Gene Expression - Post-Xenium Analyzer H&E Staining (CG000613) protocol. Images were captured using the Pannoramic 1000 (3DHISTECH).

*Custom Genes Added to the Xenium MultiTissue Panel*

**gene**

ABI3BP

ACKR3

BTK

CD55

CD248

CDH11

CLIC5

COL1A1

COMP

CXCL12

DIO2

FAP

FN1

HAS1

HBEGF

HK2

IGFBP4

IL1R1

ITGA11

LRP1

LRRC15

LRRN4CL

MALT1

MMP3

MXRA8

MYD88

NNMT

P4HB

PAPPA

PI16

PLIN2

POSTN

PTPN22

SIRPA

SLC16A1

SLC16A3

SPARC

TAB1

TNFSF11

VASN

*Visium Method*

5 µm sections of synovium were placed on an Epredia Slide following the Visium Spatial Gene Expression for FFPE – Tissue Preparation Guide (CG000518) protocol (10x Genomics).  Tissue sections were stained with hematoxylin and eosin on the Leica ST5010 auto-stainer using the same reagents and timing found in the Visium Spatial Gene Expression for FFPE – Deparaffinization, H&E Staining, Imaging & Decrosslinking (CG000520) protocol.  Images were captured using the Pannoramic 1000 by 3DHISTECH.  Once the coverslips were removed, the slides were stored at 4C° for up to 2 weeks before proceeding to decrosslinking.  The Visium Spatial Gene Expression Reagent Kits for FFPE (CG000495) protocol was used to generate probe elusion from samples.  The elution was stored at -20C before continuing onto FFPE library construction.

*Sequential Post-Xenium Bench Work*

Upon removal from the Xenium analyzer, slides were placed in Leica’s 1X Bond wash buffer and stored in the dark. Reagents from the PhenoCode Signature Immune Profile Human Protein Panel (Akoya Biosciences, Marlborough, MA, Cat #PCSPP002) were prepped for use in the assay which was run on the Leica Bond RX immunostainer. In addition to the 5 markers included in the immune profile protein panel, there is an option to include an antibody of interest; Endosialin/CD248, clone B1/35 (Millipore, Cat #MAB2626) was conjugated using Akoya’s Antibody Conjugation Kit for PhenoCode Signature (Cat #PCSP0200). Following the run, slides were placed in MilliQ water, coverslipped with Prolong Diamond Antifade Mountant (ThermoFisher, Cat #P36970) and scanned on Akoya’s PhenoImager HT. Following scanning, the autofluorescence solution, applied during the Xenium In Situ Gene Expression (CG000582), was removed using the quencher removal steps provided in Xenium In Situ Gene Expression - Post-Xenium Analyzer H&E Staining (CG000613) protocol. Using the protocol provided in CG000613, the slides were stained with hematoxylin and eosin on the Leica ST5010 auto-stainer. The slides were scanned at both 20x and 40x extended focus using the Pannoramic 1000 (3DHISTECH).   It is important to note that following scanning at every step, QC’ing was done to ensure that the images could all be aligned and could be co-registered across various platforms (Xenium Explorer, PhenoImager, and Pannoramic 1000).

***Visual QC of cell segmentation performed by Xenium***

See Xenium cell segmentation for synovium on this H&E and chromogenic IHC for coronin-1a (DAB+ brown immune cells but not fibroblasts). Automated Xenium cell segmentation algorithm performance displaying cell membrane/boundaries in white in the synovium was acceptable.

**QC of the marker genes for detecting broad cell types covered by the CosMx and Xenium single cell spatial transcriptomics platforms**

***CosMx 1K***

**Xenium MT**

**Xenium 5K**

**References**

Wang X, Wang F, Iyer AS, Knight H, Duggan LJ, Yang Y, Jin L, Cui B, He Y, Schejbal J, et al. Integrative Spatial Proteomics and Single-Cell RNA Sequencing Unveil Molecular Complexity in Rheumatoid Arthritis for Novel Therapeutic Targeting. *Proteomes* (2025) 13:17. doi: 10.3390/proteomes13020017

**Supplementary Table 1. Counts and proportions for all broad cell types by matching original CosMx FOV and spatial technologies.** Major cell type counts and proportions per matched FOV region in single cell spatial transcriptomics (CosMx-1K and Xenium MultiTissue-Custom) and mini-bulk spatial transcriptomics (Visium), split by group (normal healthy tissue, RA and OA).  Major cell type counts and proportions per matched FOV region in single cell spatial transcriptomics (CosMx-1K and Xenium MultiTissue-Custom) and mini-bulk spatial transcriptomics (Visium), split by groups (normal healthy tissue, RA and OA).   For the adipocyte annotation, we used AMP integrated with single-cell/single-nucleus reference GSE216651 (which contains 5OA and 4NH samples). The adipocyte annotation comes from 2 single-nucleus sample NH samples in GSE216651.

|  | **CosMx_FoV** | **Predicted Cell Type** | **Cell Counts** | **Proportion** | **Group** | **Technology** |
| --- | --- | --- | --- | --- | --- | --- |
| 1 | NH_A1_10_Correct | Adipose cells | 542 | 0.131937683 | NH | Xenium |
| 2 | NH_A1_10_Correct | B cells | 4 | 0.00097371 | NH | Xenium |
| 3 | NH_A1_10_Correct | CD4 T cells | 35 | 0.008519961 | NH | Xenium |
| 4 | NH_A1_10_Correct | CD8 T cells | 5 | 0.001217137 | NH | Xenium |
| 5 | NH_A1_10_Correct | Endothelial cells | 654 | 0.159201558 | NH | Xenium |
| 6 | NH_A1_10_Correct | Lining Fibroblasts | 1088 | 0.264849075 | NH | Xenium |
| 7 | NH_A1_10_Correct | Mural cells | 495 | 0.120496592 | NH | Xenium |
| 8 | NH_A1_10_Correct | Myeloid cells | 236 | 0.05744888 | NH | Xenium |
| 9 | NH_A1_10_Correct | NK_cells | 17 | 0.004138267 | NH | Xenium |
| 10 | NH_A1_10_Correct | Plasma cells | 4 | 0.00097371 | NH | Xenium |
| 11 | NH_A1_10_Correct | Sublining Fibroblasts | 1028 | 0.250243427 | NH | Xenium |
| 12 | NH_A1_15_Correct | Adipose_cells | 145 | 0.062906725 | NH | Xenium |
| 13 | NH_A1_15_Correct | CD4 T cells | 6 | 0.002603037 | NH | Xenium |
| 14 | NH_A1_15_Correct | CD8 T cells | 4 | 0.001735358 | NH | Xenium |
| 15 | NH_A1_15_Correct | Endothelial cells | 253 | 0.109761388 | NH | Xenium |
| 16 | NH_A1_15_Correct | Lining Fibroblasts | 598 | 0.259436009 | NH | Xenium |
| 17 | NH_A1_15_Correct | Mural cells | 288 | 0.12494577 | NH | Xenium |
| 18 | NH_A1_15_Correct | Myeloid cells | 228 | 0.098915401 | NH | Xenium |
| 19 | NH_A1_15_Correct | NK_cells | 5 | 0.002169197 | NH | Xenium |
| 20 | NH_A1_15_Correct | Sublining Fibroblasts | 778 | 0.337527115 | NH | Xenium |
| 21 | NH_A1_5_Correct | Adipose_cells | 208 | 0.058492688 | NH | Xenium |
| 22 | NH_A1_5_Correct | CD4 T cells | 9 | 0.002530934 | NH | Xenium |
| 23 | NH_A1_5_Correct | CD8 T cells | 4 | 0.001124859 | NH | Xenium |
| 24 | NH_A1_5_Correct | Endothelial cells | 238 | 0.066929134 | NH | Xenium |
| 25 | NH_A1_5_Correct | Lining Fibroblasts | 1534 | 0.431383577 | NH | Xenium |
| 26 | NH_A1_5_Correct | Mural cells | 418 | 0.117547807 | NH | Xenium |
| 27 | NH_A1_5_Correct | Myeloid cells | 313 | 0.088020247 | NH | Xenium |
| 28 | NH_A1_5_Correct | NK_cells | 8 | 0.002249719 | NH | Xenium |
| 29 | NH_A1_5_Correct | Sublining Fibroblasts | 824 | 0.231721035 | NH | Xenium |
| 30 | OA_A1_10 | Adipose_cells | 237 | 0.004148288 | OA | Xenium |
| 31 | OA_A1_10 | B cells | 2006 | 0.035111671 | OA | Xenium |
| 32 | OA_A1_10 | CD4 T cells | 2230 | 0.039032416 | OA | Xenium |
| 33 | OA_A1_10 | CD8 T cells | 1871 | 0.032748722 | OA | Xenium |
| 34 | OA_A1_10 | Endothelial cells | 3081 | 0.053927746 | OA | Xenium |
| 35 | OA_A1_10 | Lining Fibroblasts | 2475 | 0.043320731 | OA | Xenium |
| 36 | OA_A1_10 | Mural cells | 1427 | 0.024977246 | OA | Xenium |
| 37 | OA_A1_10 | Myeloid cells | 7412 | 0.12973465 | OA | Xenium |
| 38 | OA_A1_10 | NK_cells | 1454 | 0.025449835 | OA | Xenium |
| 39 | OA_A1_10 | Plasma cells | 29071 | 0.508839179 | OA | Xenium |
| 40 | OA_A1_10 | Sublining Fibroblasts | 5868 | 0.102709515 | OA | Xenium |
| 41 | OA_A1_15 | Adipose_cells | 98 | 0.003099991 | OA | Xenium |
| 42 | OA_A1_15 | B cells | 150 | 0.004744883 | OA | Xenium |
| 43 | OA_A1_15 | CD4 T cells | 1310 | 0.041438649 | OA | Xenium |
| 44 | OA_A1_15 | CD8 T cells | 1441 | 0.045582514 | OA | Xenium |
| 45 | OA_A1_15 | Endothelial cells | 2991 | 0.094612976 | OA | Xenium |
| 46 | OA_A1_15 | Lining Fibroblasts | 2636 | 0.083383418 | OA | Xenium |
| 47 | OA_A1_15 | Mural cells | 3590 | 0.113560877 | OA | Xenium |
| 48 | OA_A1_15 | Myeloid cells | 6744 | 0.213329959 | OA | Xenium |
| 49 | OA_A1_15 | NK_cells | 884 | 0.02796318 | OA | Xenium |
| 50 | OA_A1_15 | Plasma cells | 224 | 0.007085693 | OA | Xenium |
| 51 | OA_A1_15 | Sublining Fibroblasts | 11545 | 0.365197862 | OA | Xenium |
| 52 | OA_A1_5 | Adipose_cells | 338 | 0.021655561 | OA | Xenium |
| 53 | OA_A1_5 | B cells | 68 | 0.00435674 | OA | Xenium |
| 54 | OA_A1_5 | CD4 T cells | 218 | 0.013967196 | OA | Xenium |
| 55 | OA_A1_5 | CD8 T cells | 193 | 0.012365454 | OA | Xenium |
| 56 | OA_A1_5 | Endothelial cells | 1817 | 0.116414659 | OA | Xenium |
| 57 | OA_A1_5 | Lining Fibroblasts | 2471 | 0.158316248 | OA | Xenium |
| 58 | OA_A1_5 | Mural cells | 746 | 0.047796002 | OA | Xenium |
| 59 | OA_A1_5 | Myeloid cells | 2174 | 0.139287545 | OA | Xenium |
| 60 | OA_A1_5 | NK_cells | 262 | 0.016786263 | OA | Xenium |
| 61 | OA_A1_5 | Plasma cells | 216 | 0.013839057 | OA | Xenium |
| 62 | OA_A1_5 | Sublining Fibroblasts | 7105 | 0.455215274 | OA | Xenium |
| 63 | RA_A1_10 | Adipose_cells | 466 | 0.031867606 | RA | Xenium |
| 64 | RA_A1_10 | B cells | 231 | 0.015797032 | RA | Xenium |
| 65 | RA_A1_10 | CD4 T cells | 401 | 0.027422554 | RA | Xenium |
| 66 | RA_A1_10 | CD8 T cells | 182 | 0.012446146 | RA | Xenium |
| 67 | RA_A1_10 | Endothelial cells | 1214 | 0.0830199 | RA | Xenium |
| 68 | RA_A1_10 | Lining Fibroblasts | 2061 | 0.140942351 | RA | Xenium |
| 69 | RA_A1_10 | Mural cells | 1116 | 0.076318129 | RA | Xenium |
| 70 | RA_A1_10 | Myeloid cells | 4694 | 0.321001163 | RA | Xenium |
| 71 | RA_A1_10 | NK_cells | 314 | 0.021473022 | RA | Xenium |
| 72 | RA_A1_10 | Plasma cells | 284 | 0.019421459 | RA | Xenium |
| 73 | RA_A1_10 | Sublining Fibroblasts | 3660 | 0.250290638 | RA | Xenium |
| 74 | RA_A1_15 | Adipose_cells | 165 | 0.006230177 | RA | Xenium |
| 75 | RA_A1_15 | B cells | 2349 | 0.088695061 | RA | Xenium |
| 76 | RA_A1_15 | CD4 T cells | 2403 | 0.090734028 | RA | Xenium |
| 77 | RA_A1_15 | CD8 T cells | 1128 | 0.042591754 | RA | Xenium |
| 78 | RA_A1_15 | Endothelial cells | 2208 | 0.083371092 | RA | Xenium |
| 79 | RA_A1_15 | Lining Fibroblasts | 3567 | 0.134685093 | RA | Xenium |
| 80 | RA_A1_15 | Mural cells | 1525 | 0.057581936 | RA | Xenium |
| 81 | RA_A1_15 | Myeloid cells | 4821 | 0.182034436 | RA | Xenium |
| 82 | RA_A1_15 | NK_cells | 708 | 0.026733122 | RA | Xenium |
| 83 | RA_A1_15 | Plasma cells | 3989 | 0.150619242 | RA | Xenium |
| 84 | RA_A1_15 | Sublining Fibroblasts | 3621 | 0.13672406 | RA | Xenium |
| 85 | RA_A1_20_RA4 | Adipose_cells | 444 | 0.013602108 | RA | Xenium |
| 86 | RA_A1_20_RA4 | B cells | 4852 | 0.148642853 | RA | Xenium |
| 87 | RA_A1_20_RA4 | CD4 T cells | 2790 | 0.085472704 | RA | Xenium |
| 88 | RA_A1_20_RA4 | CD8 T cells | 1268 | 0.038845659 | RA | Xenium |
| 89 | RA_A1_20_RA4 | Endothelial cells | 1407 | 0.043103976 | RA | Xenium |
| 90 | RA_A1_20_RA4 | Lining Fibroblasts | 3223 | 0.098737822 | RA | Xenium |
| 91 | RA_A1_20_RA4 | Mural cells | 1215 | 0.037221984 | RA | Xenium |
| 92 | RA_A1_20_RA4 | Myeloid cells | 7860 | 0.240794069 | RA | Xenium |
| 93 | RA_A1_20_RA4 | NK_cells | 986 | 0.030206482 | RA | Xenium |
| 94 | RA_A1_20_RA4 | Plasma cells | 5530 | 0.169413639 | RA | Xenium |
| 95 | RA_A1_20_RA4 | Sublining Fibroblasts | 3067 | 0.093958704 | RA | Xenium |
| 96 | RA_A1_20_RA6 | Adipose_cells | 219 | 0.012371483 | RA | Xenium |
| 97 | RA_A1_20_RA6 | B cells | 767 | 0.043328437 | RA | Xenium |
| 98 | RA_A1_20_RA6 | CD4 T cells | 1426 | 0.080555869 | RA | Xenium |
| 99 | RA_A1_20_RA6 | CD8 T cells | 967 | 0.054626596 | RA | Xenium |
| 100 | RA_A1_20_RA6 | Endothelial cells | 1352 | 0.076375551 | RA | Xenium |
| 101 | RA_A1_20_RA6 | Lining Fibroblasts | 1321 | 0.074624336 | RA | Xenium |
| 102 | RA_A1_20_RA6 | Mural cells | 815 | 0.046039995 | RA | Xenium |
| 103 | RA_A1_20_RA6 | Myeloid cells | 3953 | 0.223308101 | RA | Xenium |
| 104 | RA_A1_20_RA6 | NK_cells | 621 | 0.035080782 | RA | Xenium |
| 105 | RA_A1_20_RA6 | Plasma cells | 2381 | 0.134504576 | RA | Xenium |
| 106 | RA_A1_20_RA6 | Sublining Fibroblasts | 3880 | 0.219184273 | RA | Xenium |
| 107 | RA_A1_5 | Adipose_cells | 505 | 0.022388721 | RA | Xenium |
| 108 | RA_A1_5 | B cells | 1208 | 0.053555595 | RA | Xenium |
| 109 | RA_A1_5 | CD4 T cells | 1171 | 0.051915233 | RA | Xenium |
| 110 | RA_A1_5 | CD8 T cells | 582 | 0.025802447 | RA | Xenium |
| 111 | RA_A1_5 | Endothelial cells | 1477 | 0.065481468 | RA | Xenium |
| 112 | RA_A1_5 | Lining Fibroblasts | 3600 | 0.159602766 | RA | Xenium |
| 113 | RA_A1_5 | Mural cells | 1375 | 0.06095939 | RA | Xenium |
| 114 | RA_A1_5 | Myeloid cells | 3064 | 0.135839688 | RA | Xenium |
| 115 | RA_A1_5 | NK_cells | 635 | 0.028152155 | RA | Xenium |
| 116 | RA_A1_5 | Plasma cells | 3644 | 0.161553467 | RA | Xenium |
| 117 | RA_A1_5 | Sublining Fibroblasts | 5295 | 0.234749069 | RA | Xenium |
| 118 | NH_A1_10 | Adipose_cells | 243 | 0.079672131 | NH | CosMx |
| 119 | NH_A1_10 | B cells | 14 | 0.004590164 | NH | CosMx |
| 120 | NH_A1_10 | CD4 T cells | 16 | 0.005245902 | NH | CosMx |
| 121 | NH_A1_10 | CD8 T cells | 47 | 0.015409836 | NH | CosMx |
| 122 | NH_A1_10 | Endothelial cells | 495 | 0.162295082 | NH | CosMx |
| 123 | NH_A1_10 | Lining Fibroblasts | 471 | 0.15442623 | NH | CosMx |
| 124 | NH_A1_10 | Mural cells | 426 | 0.139672131 | NH | CosMx |
| 125 | NH_A1_10 | Myeloid cells | 377 | 0.123606557 | NH | CosMx |
| 126 | NH_A1_10 | NK_cells | 39 | 0.012786885 | NH | CosMx |
| 127 | NH_A1_10 | Plasma cells | 12 | 0.003934426 | NH | CosMx |
| 128 | NH_A1_10 | Sublining Fibroblasts | 910 | 0.298360656 | NH | CosMx |
| 129 | NH_A1_15 | Adipose_cells | 189 | 0.129274966 | NH | CosMx |
| 130 | NH_A1_15 | B cells | 10 | 0.006839945 | NH | CosMx |
| 131 | NH_A1_15 | CD4 T cells | 8 | 0.005471956 | NH | CosMx |
| 132 | NH_A1_15 | CD8 T cells | 26 | 0.017783858 | NH | CosMx |
| 133 | NH_A1_15 | Endothelial cells | 279 | 0.190834473 | NH | CosMx |
| 134 | NH_A1_15 | Lining Fibroblasts | 144 | 0.098495212 | NH | CosMx |
| 135 | NH_A1_15 | Mural cells | 179 | 0.122435021 | NH | CosMx |
| 136 | NH_A1_15 | Myeloid cells | 165 | 0.112859097 | NH | CosMx |
| 137 | NH_A1_15 | NK_cells | 20 | 0.013679891 | NH | CosMx |
| 138 | NH_A1_15 | Plasma cells | 1 | 0.000683995 | NH | CosMx |
| 139 | NH_A1_15 | Sublining Fibroblasts | 441 | 0.301641587 | NH | CosMx |
| 140 | NH_A1_5 | Adipose_cells | 270 | 0.104813665 | NH | CosMx |
| 141 | NH_A1_5 | B cells | 8 | 0.00310559 | NH | CosMx |
| 142 | NH_A1_5 | CD4 T cells | 10 | 0.003881988 | NH | CosMx |
| 143 | NH_A1_5 | CD8 T cells | 32 | 0.01242236 | NH | CosMx |
| 144 | NH_A1_5 | Endothelial cells | 377 | 0.146350932 | NH | CosMx |
| 145 | NH_A1_5 | Lining Fibroblasts | 702 | 0.272515528 | NH | CosMx |
| 146 | NH_A1_5 | Mural cells | 203 | 0.078804348 | NH | CosMx |
| 147 | NH_A1_5 | Myeloid cells | 257 | 0.099767081 | NH | CosMx |
| 148 | NH_A1_5 | NK_cells | 16 | 0.00621118 | NH | CosMx |
| 149 | NH_A1_5 | Plasma cells | 1 | 0.000388199 | NH | CosMx |
| 150 | NH_A1_5 | Sublining Fibroblasts | 700 | 0.27173913 | NH | CosMx |
| 151 | OA_A1_10 | Adipose_cells | 23 | 0.000834059 | OA | CosMx |
| 152 | OA_A1_10 | B cells | 453 | 0.016427328 | OA | CosMx |
| 153 | OA_A1_10 | CD4 T cells | 194 | 0.007035103 | OA | CosMx |
| 154 | OA_A1_10 | CD8 T cells | 193 | 0.00699884 | OA | CosMx |
| 155 | OA_A1_10 | Endothelial cells | 1168 | 0.042355672 | OA | CosMx |
| 156 | OA_A1_10 | Lining Fibroblasts | 1170 | 0.042428198 | OA | CosMx |
| 157 | OA_A1_10 | Mural cells | 498 | 0.018059182 | OA | CosMx |
| 158 | OA_A1_10 | Myeloid cells | 1464 | 0.053089643 | OA | CosMx |
| 159 | OA_A1_10 | NK_cells | 71 | 0.002574703 | OA | CosMx |
| 160 | OA_A1_10 | Plasma cells | 20786 | 0.753771395 | OA | CosMx |
| 161 | OA_A1_10 | Sublining Fibroblasts | 1556 | 0.056425878 | OA | CosMx |
| 162 | OA_A1_15 | Adipose_cells | 47 | 0.002246224 | OA | CosMx |
| 163 | OA_A1_15 | B cells | 23 | 0.001099216 | OA | CosMx |
| 164 | OA_A1_15 | CD4 T cells | 89 | 0.004253489 | OA | CosMx |
| 165 | OA_A1_15 | CD8 T cells | 497 | 0.023752629 | OA | CosMx |
| 166 | OA_A1_15 | Endothelial cells | 1906 | 0.091091569 | OA | CosMx |
| 167 | OA_A1_15 | Lining Fibroblasts | 2082 | 0.099502963 | OA | CosMx |
| 168 | OA_A1_15 | Mural cells | 1292 | 0.061747276 | OA | CosMx |
| 169 | OA_A1_15 | Myeloid cells | 5315 | 0.254014529 | OA | CosMx |
| 170 | OA_A1_15 | NK_cells | 61 | 0.002915313 | OA | CosMx |
| 171 | OA_A1_15 | Plasma cells | 31 | 0.001481552 | OA | CosMx |
| 172 | OA_A1_15 | Sublining Fibroblasts | 9581 | 0.45789524 | OA | CosMx |
| 173 | OA_A1_5 | Adipose_cells | 46 | 0.004831425 | OA | CosMx |
| 174 | OA_A1_5 | B cells | 22 | 0.002310682 | OA | CosMx |
| 175 | OA_A1_5 | CD4 T cells | 31 | 0.003255961 | OA | CosMx |
| 176 | OA_A1_5 | CD8 T cells | 140 | 0.014704338 | OA | CosMx |
| 177 | OA_A1_5 | Endothelial cells | 892 | 0.093687638 | OA | CosMx |
| 178 | OA_A1_5 | Lining Fibroblasts | 961 | 0.100934776 | OA | CosMx |
| 179 | OA_A1_5 | Mural cells | 523 | 0.054931205 | OA | CosMx |
| 180 | OA_A1_5 | Myeloid cells | 1697 | 0.17823758 | OA | CosMx |
| 181 | OA_A1_5 | NK_cells | 30 | 0.00315093 | OA | CosMx |
| 182 | OA_A1_5 | Plasma cells | 272 | 0.028568428 | OA | CosMx |
| 183 | OA_A1_5 | Sublining Fibroblasts | 4907 | 0.515387039 | OA | CosMx |
| 184 | RA_A1_10 | Adipose_cells | 232 | 0.016027634 | RA | CosMx |
| 185 | RA_A1_10 | B cells | 235 | 0.016234888 | RA | CosMx |
| 186 | RA_A1_10 | CD4 T cells | 126 | 0.008704663 | RA | CosMx |
| 187 | RA_A1_10 | CD8 T cells | 364 | 0.025146805 | RA | CosMx |
| 188 | RA_A1_10 | Endothelial cells | 1551 | 0.107150259 | RA | CosMx |
| 189 | RA_A1_10 | Lining Fibroblasts | 637 | 0.044006908 | RA | CosMx |
| 190 | RA_A1_10 | Mural cells | 470 | 0.032469775 | RA | CosMx |
| 191 | RA_A1_10 | Myeloid cells | 4838 | 0.334231434 | RA | CosMx |
| 192 | RA_A1_10 | NK_cells | 125 | 0.008635579 | RA | CosMx |
| 193 | RA_A1_10 | Plasma cells | 696 | 0.048082902 | RA | CosMx |
| 194 | RA_A1_10 | Sublining Fibroblasts | 5201 | 0.359309154 | RA | CosMx |
| 195 | RA_A1_15 | Adipose_cells | 16 | 0.000813587 | RA | CosMx |
| 196 | RA_A1_15 | B cells | 1515 | 0.07703651 | RA | CosMx |
| 197 | RA_A1_15 | CD4 T cells | 563 | 0.028628089 | RA | CosMx |
| 198 | RA_A1_15 | CD8 T cells | 966 | 0.049120309 | RA | CosMx |
| 199 | RA_A1_15 | Endothelial cells | 1603 | 0.081511238 | RA | CosMx |
| 200 | RA_A1_15 | Lining Fibroblasts | 1901 | 0.096664294 | RA | CosMx |
| 201 | RA_A1_15 | Mural cells | 906 | 0.046069358 | RA | CosMx |
| 202 | RA_A1_15 | Myeloid cells | 3994 | 0.20309163 | RA | CosMx |
| 203 | RA_A1_15 | NK_cells | 90 | 0.004576426 | RA | CosMx |
| 204 | RA_A1_15 | Plasma cells | 4632 | 0.235533408 | RA | CosMx |
| 205 | RA_A1_15 | Sublining Fibroblasts | 3480 | 0.176955151 | RA | CosMx |
| 206 | RA_A1_20 | Adipose_cells | 289 | 0.005326207 | RA | CosMx |
| 207 | RA_A1_20 | B cells | 2453 | 0.045208257 | RA | CosMx |
| 208 | RA_A1_20 | CD4 T cells | 817 | 0.015057132 | RA | CosMx |
| 209 | RA_A1_20 | CD8 T cells | 2074 | 0.038223369 | RA | CosMx |
| 210 | RA_A1_20 | Endothelial cells | 3282 | 0.060486546 | RA | CosMx |
| 211 | RA_A1_20 | Lining Fibroblasts | 2772 | 0.051087357 | RA | CosMx |
| 212 | RA_A1_20 | Mural cells | 1641 | 0.030243273 | RA | CosMx |
| 213 | RA_A1_20 | Myeloid cells | 14048 | 0.258901585 | RA | CosMx |
| 214 | RA_A1_20 | NK_cells | 365 | 0.006726871 | RA | CosMx |
| 215 | RA_A1_20 | Plasma cells | 17504 | 0.322594913 | RA | CosMx |
| 216 | RA_A1_20 | Sublining Fibroblasts | 9015 | 0.166144489 | RA | CosMx |
| 217 | RA_A1_5 | Adipose_cells | 145 | 0.011537237 | RA | CosMx |
| 218 | RA_A1_5 | B cells | 285 | 0.022676639 | RA | CosMx |
| 219 | RA_A1_5 | CD4 T cells | 154 | 0.012253342 | RA | CosMx |
| 220 | RA_A1_5 | CD8 T cells | 373 | 0.029678549 | RA | CosMx |
| 221 | RA_A1_5 | Endothelial cells | 921 | 0.073281349 | RA | CosMx |
| 222 | RA_A1_5 | Lining Fibroblasts | 901 | 0.071690006 | RA | CosMx |
| 223 | RA_A1_5 | Mural cells | 516 | 0.041056652 | RA | CosMx |
| 224 | RA_A1_5 | Myeloid cells | 1814 | 0.144334819 | RA | CosMx |
| 225 | RA_A1_5 | NK_cells | 140 | 0.011139402 | RA | CosMx |
| 226 | RA_A1_5 | Plasma cells | 4800 | 0.381922342 | RA | CosMx |
| 227 | RA_A1_5 | Sublining Fibroblasts | 2519 | 0.200429663 | RA | CosMx |
| 228 | S70_CT_23031_A-1-2_OA_Synovium | Sublining Fibroblasts | - | 0.362768854 | OA | Visium |
| 229 | S70_CT_23031_A-1-2_OA_Synovium | CD4 T cells | - | 0.017886338 | OA | Visium |
| 230 | S70_CT_23031_A-1-2_OA_Synovium | Myeloid cells | - | 0.208083596 | OA | Visium |
| 231 | S70_CT_23031_A-1-2_OA_Synovium | Endothelial cells | - | 0.088924807 | OA | Visium |
| 232 | S70_CT_23031_A-1-2_OA_Synovium | Lining Fibroblasts | - | 0.160151103 | OA | Visium |
| 233 | S70_CT_23031_A-1-2_OA_Synovium | B cells | - | 0.012416453 | OA | Visium |
| 234 | S70_CT_23031_A-1-2_OA_Synovium | CD8 T cells | - | 0.005484614 | OA | Visium |
| 235 | S70_CT_23031_A-1-2_OA_Synovium | NK_cells | - | 0.003134249 | OA | Visium |
| 236 | S70_CT_23031_A-1-2_OA_Synovium | Plasma cells | - | 0.04924704 | OA | Visium |
| 237 | S70_CT_23031_A-1-2_OA_Synovium | Mural cells | - | 0.076622211 | OA | Visium |
| 238 | S73_CT_23031_A-6-2_OA_Synovium | Sublining Fibroblasts | - | 0.109569572 | OA | Visium |
| 239 | S73_CT_23031_A-6-2_OA_Synovium | CD4 T cells | - | 0.02462117 | OA | Visium |
| 240 | S73_CT_23031_A-6-2_OA_Synovium | Myeloid cells | - | 0.090314619 | OA | Visium |
| 241 | S73_CT_23031_A-6-2_OA_Synovium | Endothelial cells | - | 0.072958757 | OA | Visium |
| 242 | S73_CT_23031_A-6-2_OA_Synovium | Lining Fibroblasts | - | 0.022975862 | OA | Visium |
| 243 | S73_CT_23031_A-6-2_OA_Synovium | B cells | - | 0.012522292 | OA | Visium |
| 244 | S73_CT_23031_A-6-2_OA_Synovium | CD8 T cells | - | 0.006171984 | OA | Visium |
| 245 | S73_CT_23031_A-6-2_OA_Synovium | NK_cells | - | 0.002439305 | OA | Visium |
| 246 | S73_CT_23031_A-6-2_OA_Synovium | Plasma cells | - | 0.602227183 | OA | Visium |
| 247 | S73_CT_23031_A-6-2_OA_Synovium | Mural cells | - | 0.040600645 | OA | Visium |
| 248 | S75_CT_19060_D-8-2_Normal_Synovium | Sublining Fibroblasts | - | 0.357661831 | NH | Visium |
| 249 | S75_CT_19060_D-8-2_Normal_Synovium | CD4 T cells | - | 0.013018577 | NH | Visium |
| 250 | S75_CT_19060_D-8-2_Normal_Synovium | Myeloid cells | - | 0.171218001 | NH | Visium |
| 251 | S75_CT_19060_D-8-2_Normal_Synovium | Endothelial cells | - | 0.187535274 | NH | Visium |
| 252 | S75_CT_19060_D-8-2_Normal_Synovium | Lining Fibroblasts | - | 0.100474918 | NH | Visium |
| 253 | S75_CT_19060_D-8-2_Normal_Synovium | B cells | - | 0.008442573 | NH | Visium |
| 254 | S75_CT_19060_D-8-2_Normal_Synovium | CD8 T cells | - | 0.003212379 | NH | Visium |
| 255 | S75_CT_19060_D-8-2_Normal_Synovium | NK_cells | - | 0.00272467 | NH | Visium |
| 256 | S75_CT_19060_D-8-2_Normal_Synovium | Plasma cells | - | 0.001938778 | NH | Visium |
| 257 | S75_CT_19060_D-8-2_Normal_Synovium | Mural cells | - | 0.138794036 | NH | Visium |
| 258 | S76_CT_23031_A-8-3_OA_Synovium | Sublining Fibroblasts | - | 0.321471172 | OA | Visium |
| 259 | S76_CT_23031_A-8-3_OA_Synovium | CD4 T cells | - | 0.030346122 | OA | Visium |
| 260 | S76_CT_23031_A-8-3_OA_Synovium | Myeloid cells | - | 0.189322243 | OA | Visium |
| 261 | S76_CT_23031_A-8-3_OA_Synovium | Endothelial cells | - | 0.193689303 | OA | Visium |
| 262 | S76_CT_23031_A-8-3_OA_Synovium | Lining Fibroblasts | - | 0.073112027 | OA | Visium |
| 263 | S76_CT_23031_A-8-3_OA_Synovium | B cells | - | 0.011045381 | OA | Visium |
| 264 | S76_CT_23031_A-8-3_OA_Synovium | CD8 T cells | - | 0.006755226 | OA | Visium |
| 265 | S76_CT_23031_A-8-3_OA_Synovium | NK_cells | - | 0.002601013 | OA | Visium |
| 266 | S76_CT_23031_A-8-3_OA_Synovium | Plasma cells | - | 0.006530182 | OA | Visium |
| 267 | S76_CT_23031_A-8-3_OA_Synovium | Mural cells | - | 0.144850673 | OA | Visium |
| 268 | S77_CT_21045_A-15-3_RA_Synovium | Sublining Fibroblasts | - | 0.262823316 | RA | Visium |
| 269 | S77_CT_21045_A-15-3_RA_Synovium | CD4 T cells | - | 0.061379746 | RA | Visium |
| 270 | S77_CT_21045_A-15-3_RA_Synovium | Myeloid cells | - | 0.230065517 | RA | Visium |
| 271 | S77_CT_21045_A-15-3_RA_Synovium | Endothelial cells | - | 0.125403463 | RA | Visium |
| 272 | S77_CT_21045_A-15-3_RA_Synovium | Lining Fibroblasts | - | 0.067266373 | RA | Visium |
| 273 | S77_CT_21045_A-15-3_RA_Synovium | B cells | - | 0.04342846 | RA | Visium |
| 274 | S77_CT_21045_A-15-3_RA_Synovium | CD8 T cells | - | 0.011603084 | RA | Visium |
| 275 | S77_CT_21045_A-15-3_RA_Synovium | NK_cells | - | 0.004598542 | RA | Visium |
| 276 | S77_CT_21045_A-15-3_RA_Synovium | Plasma cells | - | 0.082989833 | RA | Visium |
| 277 | S77_CT_21045_A-15-3_RA_Synovium | Mural cells | - | 0.081547423 | RA | Visium |
| 278 | S78_CT_513_A-1-1_RA_Synovium | Sublining Fibroblasts | - | 0.091117876 | RA | Visium |
| 279 | S78_CT_513_A-1-1_RA_Synovium | CD4 T cells | - | 0.057635365 | RA | Visium |
| 280 | S78_CT_513_A-1-1_RA_Synovium | Myeloid cells | - | 0.273290336 | RA | Visium |
| 281 | S78_CT_513_A-1-1_RA_Synovium | Endothelial cells | - | 0.04825271 | RA | Visium |
| 282 | S78_CT_513_A-1-1_RA_Synovium | Lining Fibroblasts | - | 0.158025644 | RA | Visium |
| 283 | S78_CT_513_A-1-1_RA_Synovium | B cells | - | 0.059742267 | RA | Visium |
| 284 | S78_CT_513_A-1-1_RA_Synovium | CD8 T cells | - | 0.010468558 | RA | Visium |
| 285 | S78_CT_513_A-1-1_RA_Synovium | NK_cells | - | 0.002990203 | RA | Visium |
| 286 | S78_CT_513_A-1-1_RA_Synovium | Plasma cells | - | 0.232963553 | RA | Visium |
| 287 | S78_CT_513_A-1-1_RA_Synovium | Mural cells | - | 0.04143587 | RA | Visium |
| 288 | S79_CT_514_A-1-3_RA_Synovium | Sublining Fibroblasts | - | 0.174964348 | RA | Visium |
| 289 | S79_CT_514_A-1-3_RA_Synovium | CD4 T cells | - | 0.028648413 | RA | Visium |
| 290 | S79_CT_514_A-1-3_RA_Synovium | Myeloid cells | - | 0.204044399 | RA | Visium |
| 291 | S79_CT_514_A-1-3_RA_Synovium | Endothelial cells | - | 0.111673755 | RA | Visium |
| 292 | S79_CT_514_A-1-3_RA_Synovium | Lining Fibroblasts | - | 0.227519182 | RA | Visium |
| 293 | S79_CT_514_A-1-3_RA_Synovium | B cells | - | 0.023711484 | RA | Visium |
| 294 | S79_CT_514_A-1-3_RA_Synovium | CD8 T cells | - | 0.005421486 | RA | Visium |
| 295 | S79_CT_514_A-1-3_RA_Synovium | NK_cells | - | 0.00340307 | RA | Visium |
| 296 | S79_CT_514_A-1-3_RA_Synovium | Plasma cells | - | 0.106316595 | RA | Visium |
| 297 | S79_CT_514_A-1-3_RA_Synovium | Mural cells | - | 0.081544336 | RA | Visium |
| 298 | S80_CT_19060_A-4-1_RA_Synovium | Sublining Fibroblasts | - | 0.170537836 | RA | Visium |
| 299 | S80_CT_19060_A-4-1_RA_Synovium | CD4 T cells | - | 0.062227923 | RA | Visium |
| 300 | S80_CT_19060_A-4-1_RA_Synovium | Myeloid cells | - | 0.289293348 | RA | Visium |
| 301 | S80_CT_19060_A-4-1_RA_Synovium | Endothelial cells | - | 0.059784525 | RA | Visium |
| 302 | S80_CT_19060_A-4-1_RA_Synovium | Lining Fibroblasts | - | 0.118024618 | RA | Visium |
| 303 | S80_CT_19060_A-4-1_RA_Synovium | B cells | - | 0.018761834 | RA | Visium |
| 304 | S80_CT_19060_A-4-1_RA_Synovium | CD8 T cells | - | 0.019485323 | RA | Visium |
| 305 | S80_CT_19060_A-4-1_RA_Synovium | NK_cells | - | 0.005191162 | RA | Visium |
| 306 | S80_CT_19060_A-4-1_RA_Synovium | Plasma cells | - | 0.188955908 | RA | Visium |
| 307 | S80_CT_19060_A-4-1_RA_Synovium | Mural cells | - | 0.037838379 | RA | Visium |

**Supplementary Table 2. Correlation analyses on major cell type proportions across spatial technologies.** Major cell type counts and proportions per matched FOV region in single cell spatial transcriptomics (CosMx-1K and Xenium MultiTissue-Custom) and mini-bulk spatial transcriptomics (Visium), split by group (normal healthy tissue, RA and OA).  Major cell type counts and proportions per matched FOV region in single cell spatial transcriptomics (CosMx-1K and Xenium MultiTissue-Custom) and mini-bulk spatial transcriptomics (Visium), split by groups (normal healthy tissue, RA and OA).

|  | **CosMx** | **Visium** | **Xenium** |
| --- | --- | --- | --- |
| NH_A1_10_B cells | 0.004590164 | NA | 0.00097371 |
| NH_A1_10_CD4 T cells | 0.005245902 | NA | 0.008519961 |
| NH_A1_10_CD8 T cells | 0.015409836 | NA | 0.001217137 |
| NH_A1_10_Endothelial cells | 0.162295082 | NA | 0.159201558 |
| NH_A1_10_Lining Fibroblasts | 0.15442623 | NA | 0.264849075 |
| NH_A1_10_Mural cells | 0.139672131 | NA | 0.120496592 |
| NH_A1_10_Myeloid cells | 0.123606557 | NA | 0.05744888 |
| NH_A1_10_Plasma cells | 0.003934426 | NA | 0.00097371 |
| NH_A1_10_Sublining Fibroblasts | 0.298360656 | NA | 0.250243427 |
| NH_A1_15_B cells | 0.006839945 | 0.008442573 | NA |
| NH_A1_15_CD4 T cells | 0.005471956 | 0.013018577 | 0.002603037 |
| NH_A1_15_CD8 T cells | 0.017783858 | 0.003212379 | 0.001735358 |
| NH_A1_15_Endothelial cells | 0.190834473 | 0.187535274 | 0.109761388 |
| NH_A1_15_Lining Fibroblasts | 0.098495212 | 0.100474918 | 0.259436009 |
| NH_A1_15_Mural cells | 0.122435021 | 0.138794036 | 0.12494577 |
| NH_A1_15_Myeloid cells | 0.112859097 | 0.171218001 | 0.098915401 |
| NH_A1_15_Plasma cells | 0.000683995 | 0.001938778 | NA |
| NH_A1_15_Sublining Fibroblasts | 0.301641587 | 0.357661831 | 0.337527115 |
| NH_A1_5_B cells | 0.00310559 | NA | NA |
| NH_A1_5_CD4 T cells | 0.003881988 | NA | 0.002530934 |
| NH_A1_5_CD8 T cells | 0.01242236 | NA | 0.001124859 |
| NH_A1_5_Endothelial cells | 0.146350932 | NA | 0.066929134 |
| NH_A1_5_Lining Fibroblasts | 0.272515528 | NA | 0.431383577 |
| NH_A1_5_Mural cells | 0.078804348 | NA | 0.117547807 |
| NH_A1_5_Myeloid cells | 0.099767081 | NA | 0.088020247 |
| NH_A1_5_Plasma cells | 0.000388199 | NA | NA |
| NH_A1_5_Sublining Fibroblasts | 0.27173913 | NA | 0.231721035 |
| OA_A1_10_B cells | 0.016427328 | 0.012522292 | 0.035111671 |
| OA_A1_10_CD4 T cells | 0.007035103 | 0.02462117 | 0.039032416 |
| OA_A1_10_CD8 T cells | 0.00699884 | 0.006171984 | 0.032748722 |
| OA_A1_10_Endothelial cells | 0.042355672 | 0.072958757 | 0.053927746 |
| OA_A1_10_Lining Fibroblasts | 0.042428198 | 0.022975862 | 0.043320731 |
| OA_A1_10_Mural cells | 0.018059182 | 0.040600645 | 0.024977246 |
| OA_A1_10_Myeloid cells | 0.053089643 | 0.090314619 | 0.12973465 |
| OA_A1_10_Plasma cells | 0.753771395 | 0.602227183 | 0.508839179 |
| OA_A1_10_Sublining Fibroblasts | 0.056425878 | 0.109569572 | 0.102709515 |
| OA_A1_15_B cells | 0.001099216 | 0.011045381 | 0.004744883 |
| OA_A1_15_CD4 T cells | 0.004253489 | 0.030346122 | 0.041438649 |
| OA_A1_15_CD8 T cells | 0.023752629 | 0.006755226 | 0.045582514 |
| OA_A1_15_Endothelial cells | 0.091091569 | 0.193689303 | 0.094612976 |
| OA_A1_15_Lining Fibroblasts | 0.099502963 | 0.073112027 | 0.083383418 |
| OA_A1_15_Mural cells | 0.061747276 | 0.144850673 | 0.113560877 |
| OA_A1_15_Myeloid cells | 0.254014529 | 0.189322243 | 0.213329959 |
| OA_A1_15_Plasma cells | 0.001481552 | 0.006530182 | 0.007085693 |
| OA_A1_15_Sublining Fibroblasts | 0.45789524 | 0.321471172 | 0.365197862 |
| OA_A1_5_B cells | 0.002310682 | 0.012416453 | 0.00435674 |
| OA_A1_5_CD4 T cells | 0.003255961 | 0.017886338 | 0.013967196 |
| OA_A1_5_CD8 T cells | 0.014704338 | 0.005484614 | 0.012365454 |
| OA_A1_5_Endothelial cells | 0.093687638 | 0.088924807 | 0.116414659 |
| OA_A1_5_Lining Fibroblasts | 0.100934776 | 0.160151103 | 0.158316248 |
| OA_A1_5_Mural cells | 0.054931205 | 0.076622211 | 0.047796002 |
| OA_A1_5_Myeloid cells | 0.17823758 | 0.208083596 | 0.139287545 |
| OA_A1_5_Plasma cells | 0.028568428 | 0.04924704 | 0.013839057 |
| OA_A1_5_Sublining Fibroblasts | 0.515387039 | 0.362768854 | 0.455215274 |
| RA_A1_10_B cells | 0.016234888 | NA | 0.015797032 |
| RA_A1_10_CD4 T cells | 0.008704663 | NA | 0.027422554 |
| RA_A1_10_CD8 T cells | 0.025146805 | NA | 0.012446146 |
| RA_A1_10_Endothelial cells | 0.107150259 | NA | 0.0830199 |
| RA_A1_10_Lining Fibroblasts | 0.044006908 | NA | 0.140942351 |
| RA_A1_10_Mural cells | 0.032469775 | NA | 0.076318129 |
| RA_A1_10_Myeloid cells | 0.334231434 | NA | 0.321001163 |
| RA_A1_10_Plasma cells | 0.048082902 | NA | 0.019421459 |
| RA_A1_10_Sublining Fibroblasts | 0.359309154 | NA | 0.250290638 |
| RA_A1_15_B cells | 0.07703651 | 0.04342846 | 0.088695061 |
| RA_A1_15_CD4 T cells | 0.028628089 | 0.061379746 | 0.090734028 |
| RA_A1_15_CD8 T cells | 0.049120309 | 0.011603084 | 0.042591754 |
| RA_A1_15_Endothelial cells | 0.081511238 | 0.125403463 | 0.083371092 |
| RA_A1_15_Lining Fibroblasts | 0.096664294 | 0.067266373 | 0.134685093 |
| RA_A1_15_Mural cells | 0.046069358 | 0.081547423 | 0.057581936 |
| RA_A1_15_Myeloid cells | 0.20309163 | 0.230065517 | 0.182034436 |
| RA_A1_15_Plasma cells | 0.235533408 | 0.082989833 | 0.150619242 |
| RA_A1_15_Sublining Fibroblasts | 0.176955151 | 0.262823316 | 0.13672406 |
| RA_A1_20_B cells | 0.045208257 | 0.034071862 | 0.095985645 |
| RA_A1_20_CD4 T cells | 0.015057132 | 0.0495039 | 0.083014287 |
| RA_A1_20_CD8 T cells | 0.038223369 | 0.011791789 | 0.046736128 |
| RA_A1_20_Endothelial cells | 0.060486546 | 0.073236997 | 0.059739764 |
| RA_A1_20_Lining Fibroblasts | 0.051087357 | 0.167856481 | 0.086681079 |
| RA_A1_20_Mural cells | 0.030243273 | 0.053606195 | 0.04163099 |
| RA_A1_20_Myeloid cells | 0.258901585 | 0.255542694 | 0.232051085 |
| RA_A1_20_Plasma cells | 0.322594913 | 0.176078685 | 0.151959108 |
| RA_A1_20_Sublining Fibroblasts | 0.166144489 | 0.14554002 | 0.156571489 |
| RA_A1_5_B cells | 0.022676639 | NA | 0.053555595 |
| RA_A1_5_CD4 T cells | 0.012253342 | NA | 0.051915233 |
| RA_A1_5_CD8 T cells | 0.029678549 | NA | 0.025802447 |
| RA_A1_5_Endothelial cells | 0.073281349 | NA | 0.065481468 |
| RA_A1_5_Lining Fibroblasts | 0.071690006 | NA | 0.159602766 |
| RA_A1_5_Mural cells | 0.041056652 | NA | 0.06095939 |
| RA_A1_5_Myeloid cells | 0.144334819 | NA | 0.135839688 |
| RA_A1_5_Plasma cells | 0.381922342 | NA | 0.161553467 |
| RA_A1_5_Sublining Fibroblasts | 0.200429663 | NA | 0.234749069 |

**Supplementary Table 3. Alignment of reported fibroblast populations of the RA synovium based on the five top genes expressed.**

| **Fibroblast population (defined by AMP)** | **Top genes CosMx Fibroblast Annotations** |
| --- | --- |
| **Lining** | **PRG4**  **CLU**  **MMP3**  **MMP1**  **TNFAIP6** |
| **Mural (pericytes)** | **IGFBP7**  **NOTCH3**  **TAGLN**  **ACTA2**  **RGS5** |
| **RSPO+** | **MT2A**  **VCAM1**  **TNXA/B1**  **GSN**  **MRC2** |
| **CXC12+** | **DCN**  **CXCL12**  **LUM**  **IGFBP41**  **MMP2** |
| **CD74+** | **THBS21**  **MXRA8**  **CCL2**  **IGFBP4**  **RARRES21** |
| **NOTCH3+** | **CXCL14**  **APOD**  **COL6A1**  **VCAN**  **COL5A3** |
| **POSTN+** | **COL1A2**  **COL1A1**  **COL6A3**  **COMP**  **COL14A1** |
| **DKK3+** | **IGFBP6**  **MFAP53**  **PTGIS1**  **SPARC**  **COL5A11** |
| **CD34+** | **PI16**  **MFAP5**  **VEGFD1**  **CD70**  **PTGIS** |

**Supplementary Figure 1.  Final Sample Datasets for Each Modality Based on Quality Control**

A. Pathologist evaluation of synovial samples ensures good tissue architecture and size, good quality of histological morphology and representative compartments of the synovium. B. Following quality control checks, including an H+E, spatial transcriptomic technologies, CosMx (human 1K panel), Xenium (human MultiT-custom panel), Visium and Laser Capture Microscopy (LCM) proteomics were applied to serial sections of normal healthy (NH) (n=3) and rheumatoid arthritis (RA) (n=6) synovial tissue. In addition, using a same-slide approach, one NH and four RA samples were run on the Xenium analyzer (human 5K panel), stained with Akoya’s PhenoCode Signature Immune Profile Human Protein Panel, plus CD248, and lastly, stained with an H+E. This approach allowed for comparison across technologies as well as between disease and healthy states to gain a more complete understanding of the spatial and molecular interactions in the synovium.

 A.

B.

**Supplementary Figure 2. Broad and extended cell type annotations in scST technologies allow for comparison of cell types and differential gene expression.**

A. Comparison of major cell type maps between a CosMx and Xenium FOV from a single RA sample (serial sections). Visium heat map spot plots after RCTD deconvolution displayed the topography of several major cell types in the same RA sample (serial section). A healthy sample has been added to show the increase of cell counts in disease. Qualitative comparisons between the localization of Xenium and Visium for each specific cell type aid in the verification of cell type annotations in Visium B. Correlation between cell type proportions using annotations across spatial transcriptomic platforms across CosMx FOVs (from 3 healthy, 3 OA and 5 RA samples) and same FOV’s transferred to Xenium and Visium image sets. C. UMAP representation of major cell types using CosMx cell type annotations in the 3 healthy, 3 OA and 5 RA samples. D. Correlation between log fold changes in disease vs healthy synovium, by technology pair.

**A**

 

**B**

**C**

 

**D.**

**Supplementary Figure 3. Multimodal spatial omics: protein and RNA-Xenium heatmaps on 14 representative overlapping fibroblast marker features.**

(A) Example of pathologists’ LCM annotations of the RA synovial compartments on H&E stained sections: Lining (red), Immune SSL (green) and Fibrous SSL (blue). (B) Transfer of H&E LCM ROIs from 3 healthy and 6 RA samples to the Xenium corresponding sample set in BioTuring for pseudo bulk analysis. (C) Protein expression heatmap of 14 overlapping fibroblast marker features in RA compartments. (D) mRNA expression heatmap of 14 overlapping fibroblast marker features in RA compartments. Protein and RNA expression heatmap are normalized based on each feature or row.

**Supplementary Figure 4. Pairwise fold change comparisons in 30 overlapping fibroblast features between pseudo-bulk spatial transcriptomics-Xenium and LCM-proteomics in 3 healthy and 6 RA synovial samples. (A)** The schematic shows 7 pairwise comparisons among 5 pathological regions: NH SL, NH SSL, RA SL, RA fibrous SSL and RA immune SSL. The arrows labeling with 2, 6 and 7 numbers indicate 3 cross-disease-state comparisons, while the arrows labeling with 1, 8, 9, and 10 indicate 4 intra-disease-state comparisons. **(B-H)** The scatter plots show the differential expression of 14 fibroblast cell markers comparing mRNA and protein in 7 region-to-region comparisons. 3 cross-disease-state comparisons in **(B-D)** and 4 intra-disease-state comparisons in **(E-H)**. Blue and green dots indicate features with the same mRNA and protein expression directionality. Orange dots indicate features with different directionality. Pearson correlation coefficients are also listed in each panel.

**Supplementary Figure 5. Limitations, challenges and recommendations of spatial work in synovium**. **(A)** The cellular composition of the synovium can depend on the depth of the section. Thus, if we compare two sections from the same biopsied sample that are 80 microns apart, we can still detect the presence of all the fibroblast subtypes, but the proportions can vary. **(B)** The sublining of the synovium can be composed of various mesenchymal types of tissue. The fibroblast composition of the synovium can be biased by the type of tissue that is present in the sublining. In this synovial biopsy from a subject with osteoarthritis, the fibroblast subtypes represented in the area with fibrous connective tissue are different from those subtypes represented in adipose tissue. **(C)** Spatial plots displaying fibroblast subtypes in the representative Normal (NH1) and RA (RA3) disease sample. (D) Heatmaps of Pearson correlation coefficients between fibroblast neighborhoods across all normal and RA samples. The magnitude of the correlation coefficient corresponds to the color scale of the heatmap, ranging from blue (-1) to white (0) to red (1).

**C**

D

 
